# Development of a Collagen–Cerium Oxide Nanohydrogel for Wound Healing: In Vitro and In Vivo Evaluation

**DOI:** 10.3390/biomedicines13112623

**Published:** 2025-10-26

**Authors:** Ekaterina Vladimirovna Silina, Natalia Evgenievna Manturova, Victor Ivanovich Sevastianov, Nadezhda Victorovna Perova, Mikhail Petrovich Gladchenko, Alexey Anatolievich Kryukov, Aleksandr Victorovich Ivanov, Victor Tarasovich Dudka, Evgeniya Valerievna Prazdnova, Sergey Alexandrovich Emelyantsev, Evgenia Igorevna Kozhukhova, Vladimir Anatolievich Parfenov, Alexander Vladimirovich Ivanov, Mikhail Alexandrovich Popov, Victor Alexandrovich Stupin

**Affiliations:** 1 I.M. Sechenov First Moscow State Medical University (Sechenov University), Moscow 119991, Russia; parfenov_v_a@staff.sechenov.ru; 2 Pirogov Russian National Research Medical University, Moscow 117997, Russia; manturovanatali@yandex.ru (N.E.M.); stvictor@bk.ru (V.A.S.); 3 Institute of Biomedical Research and Technology, Moscow 123557, Russia; viksev@yandex.ru (V.I.S.); post@imbiit.ru (N.V.P.); 4 Kursk State Medical University, Kursk 305041, Russia; mgladchenko@yandex.ru (M.P.G.); krukovaa@kursksmu.net (A.A.K.); ivanovav@kursksmu.net (A.V.I.); dudkavt@kursksmu.net (V.T.D.); 5 Southern Federal University, Rostov-on-Don 344090, Russia; prazdnova@sfedu.ru (E.V.P.); emelyancev@sfedu.ru (S.A.E.); 6 National Research Center “Kurchatov Institute”, Moscow 123182, Russia; evgeshapanarina@gmail.com; 7 Institute of General Pathology and Pathophysiology, Moscow 125315, Russia; ivanov_av82@mail.ru (A.V.I.); popovcardio88@mail.ru (M.A.P.)

**Keywords:** collagen, hydrogel, cerium oxide, nanoparticles, composites, nanocerium, biosensor, wound healing, fibroblasts, leukocytes, rats, antioxidants, regenerative drug

## Abstract

**Background**: Effective regenerative therapeutics for acute and chronic wounds remain a critical unmet need in biomedicine. **Objectives**: This study aimed to develop novel collagen–cerium oxide nanoparticle hydrogels designed to enhance cellular metabolism, proliferation, and antioxidant/antimutagenic activity, accelerating wound *regeneration* in vivo. **Methods**: Collagen–nanocerium composites were synthesized by combining a collagen extract with cerium oxide nanoparticles at defined concentrations. In vitro assays using human fibroblasts identified two formulations that enhanced proliferation and metabolic activity by 42–50%. FTIR spectroscopy confirmed chemical interactions within the composite matrix. Toxicity, antioxidant, and antigenotoxic effects were evaluated using *Escherichia coli* MG1655 lux-biosensors to assess their general toxicity, antioxidant and pro-oxidant activities, and antigenotoxic and promutagenic effects. In vivo efficacy was tested in Wistar rats with full-thickness skin wounds. Treated groups were compared to untreated controls and Dexpanthenol-treated positive controls. On days 3, 7, and 14, healing was assessed clinically, histologically, and morphometrically. **Results**: Biosensor analysis demonstrated non-toxicity and antigenotoxic activity of the nanocomposites, reduced DNA damage by up to 45%, providing 31–49% protection against H_2_O_2_ and 15–23% against O_2_^−^ radicals. The animal study results demonstrated significantly accelerated healing with both nanocomposites versus control and comparison groups, evidenced by improved tissue regeneration, reduced inflammation, and increased fibroblast infiltration. **Conclusions**: The developed hydrogels exhibit promising pharmacological profiles, including antioxidant, antimutagenic, anti-inflammatory, and pro-regenerative effects validated across in vitro and in vivo models.

## 1. Introduction

Management of skin wounds and ulcers remains a highly relevant challenge, as pharmacological and surgical interventions often fail to provide complete resolution. The volume of PubMed publications on this subject has remained consistently high, with 11,309 articles published over the past 5 years and 20,558 over the past decade. The number of studies focused on developing new therapeutic drugs is also increasing; a search for “skin wounds” and “treatment results” has yielded 653 studies in the past 5 years. This trend underscores the persistent relevance of the problem. Treatment of patients with chronic wounds associated with arterial and venous lower limb vascular anomalies, type 2 diabetes mellitus, and peripheral neuropathy is particularly challenging [1,2,3,4]. Driven by a remarkable increase in life expectancy and prevalence of comorbidities, the number of these patients is rapidly increasing [5,6,7,8]. This situation imposes a significant social and financial burden on health care budgets at all levels.

The application of collagen as a component of wound dressings or in injectable forms has previously demonstrated favorable outcomes, although not in all cases [9,10,11,12,13,14,15,16,17]. Collagen is a triple-helical glycoprotein and fibrillar protein that is crucial for the structure and function of connective tissues and provides them with strength and elasticity. Fibroblasts (specialized connective tissue cells) secrete collagen to create protein scaffolds that are essential for the normal functioning of other cells under normal physiological conditions [17,18,19,20]. Collagen can be extracted from various animal-derived, collagen-rich tissues such as cartilage, skin, and bones, which are by-products of food processing technologies [21,22,23,24,25] and are frequently destined for disposal. The mechanism of collagen regeneration is complex and has not yet been fully elucidated. Collagen serves as the basic building block of a native extracellular matrix (ECM) for the directed migration of mesenchymal stem cells, fibroblasts, and macrophages into the wound bed. This pathway accelerates the appearance of proregenerative M2 macrophages in the wound, reduces inflammation severity, and alters the expression profile of factors involved in wound healing [26,27,28,29,30,31]. Unfortunately, collagen-based compositions have not proven to be a panacea for wound management, and the search for new forms and drugs for wound healing has continued.

Among the new promising avenues that have emerged over the last 10–20 years, significant interest has been focused on nanomaterials, which are engineered using nanoparticles or nanotechnology that often possess unique properties that are beneficial for wound regeneration. Numerous studies on the potential applications of nanomaterials in biology and medicine have focused on cerium dioxide nanoparticles (nanocerium), which are lanthanide nanoparticles with an atomic number of 58 [32,33,34,35,36,37,38].

Nanoparticles are conventionally defined as objects ranging in size from 1 to 1000 nm; however, their biological effects are typically observed within a narrow range of 1–100 nm. Nanomedicine, which has emerged as a distinct branch of medical science, focuses on the application of nanotechnology and related research for advancements in human and veterinary medicine. In biological systems, the effective size range for nanoparticles generally lies between approximately 1 nm, corresponding to clusters of 3–5 atoms that retain the properties of a heterogeneous colloidal environment, and an upper limit of 20–25 nm, beyond which efficacy often diminishes.

Nature has provided well-established examples of nanoscale biological regulatory systems. These systems first appeared in the earliest living cells, and included molecular mechanisms responsible for RNA and protein replication, highly sensitive biosensors, and other nanoscale structures that facilitate survival in a hostile primordial environment.

Currently, nanomedicine is primarily employed in three major domains: nanodiagnostics (e.g., as contrast agents for enhanced imaging in computed tomography), nanotherapy (including targeted drug delivery to tumor cells), and regenerative medicine, particularly in the development of scaffolds to replace extensive soft tissue or bone defects. Metal-based nanoparticles, especially those containing elements with variable valence states, have demonstrated beneficial biological properties, including the ability to modulate redox processes within the cellular microenvironment. This is exactly what cerium oxide nanoparticles are.

This interest is readily explained by the fact that cerium oxide (CeO_2_) nanoparticles have demonstrated exceptional biological properties, including antibacterial, anti-inflammatory, antioxidant, and pro-angiogenic effects [39,40,41,42,43]. The beneficial biological effects of using cerium oxide nanoparticles are so obvious that an increasing number of researchers are beginning to work on the possible medical applications of nanoceria, including stroke, diabetes, cardiomyopathy, and regenerative medicine in general [33,36,41,42,43,44,45,46,47,48].

However, the strong propensity of nanoparticles to agglomerate owing to van der Waals forces is a significant challenge, which substantially diminishes their beneficial biological properties [49,50]. In this regard, there is a global research effort to investigate reliable synthesis methods for nanocrystalline ceria coated with various excipients, particularly polysaccharides, carboxylic acids, and polymers [51]. Such nanocomposites show no signs of agglomeration, while simultaneously enhancing the beneficial biological properties of the nanoparticles.

Given the multifunctional properties of the ECM, recent years have witnessed a growing research interest in multicomponent biopolymeric hydrogels, biomimetics of ESM, which incorporate all major ECM components (glycoproteins, proteoglycans, and hyaluronates), as well as growth factors and other signaling molecules essential for cell adhesion, proliferation, and differentiation [52]. Biopolymeric hydrogels are more effective in supporting cell adhesion, proliferation, and differentiation than mono- and bicomponent hydrogels containing only one (e.g., collagen or hyaluronic acid) or two (e.g., collagen and hyaluronic acid) ECM components [53,54,55,56]. We hypothesized that the combination of collagen and nanocerium, which differ in their mechanisms of tissue regeneration enhancement, could yield a synergistic effect and potentially lead to the development of a novel and highly effective drug for acute and chronic wound management.

This study aimed to conduct a comprehensive biomedical investigation to select the optimal composition of a novel medical drug for wound management based on cerium dioxide nanoparticles and an ECM-mimetic, hydrogel collagen-containing scaffold that enhances cellular metabolism and proliferation while also exhibiting antimutagenic and antioxidant effects. Ultimately, this line of research is expected to result in accelerated and high-quality wound healing in mammals.

To achieve this goal, the following tasks were solved:

1. To develop the synthesis of new collagen-nanoceria composites with new pharmacological and regenerative properties.
2. To conduct cultural studies and select the best components for the effectiveness and safety of the nanocomposites.
3. To obtain data on the induction and protective activity for parameters such as general toxicity, antioxidant, prooxidant, antigenotoxic, and promutagenic activity, using live bacterial biosensors.
4. To study the effectiveness and regenerative potential of the developed nanocompositions on full-thickness skin wound models in animals.

## 2. Materials and Methods

This interdisciplinary study was conducted through the collaborative effort of various specialists in biology, medicine, chemistry, physics, and pharmacy across different establishments within the Russian Federation. All stages of the medical regenerative product development study were conducted in close collaboration with healthcare professionals from various specialties, mainly general and plastic surgeons, who are the primary end users of the wound healing product.

### 2.1. Study Design

The study used two types of cerium dioxide nanoparticles (nanoceria) stabilized with citric acid (CeO-CA) and dextran (CeO-Dex), synthesized according to the technology detailed in our previous research [57,58,59], at three concentrations: 10^−2^ M, 10^−3^ M, and 10^−4^ M. Collagen extract was prepared and serially diluted (100%, 50%, 25%, 20%, 16.7%, and 10%). Subsequently, nanocomposites were formulated based on selected concentrations of collagen and nanoceria. Fourier-transform infrared (FTIR) spectroscopy was used to assess the compositional composition of the collagen–nanocerium composites.

An initial series of experiments was conducted on the human fibroblast cell line BJ hTERT (American Type Culture Collection, ATCC, CRL-3627, Manassas, VA, USA; source: newborn foreskin) to determine the most effective and safe composition and concentrations of the developed nanodrug. Optimal concentrations of collagen–nanocerium composites that maximally stimulated key cells involved in skin wound regeneration were identified. Based on these data, collagen–nanocerium composite complexes containing the optimal concentration of the studied substances were developed and subsequently re-tested on the BJ hTERT cell line.

Following this experimental series, two prototype regenerative collagen–nanocerium drugs were selected from multiple variants for further investigation in living systems using bacterial biosensors and in an animal model of acute full-thickness skin wounds.

The selected nanocomposite prototypes were analyzed using several bacterial biosensors and genetically modified *Escherichia coli* (*E. coli*) to assess their safety (toxicity), antimutagenicity, and redox activity.

After demonstrating the safety and efficacy of the composites in vitro, experimental in vivo studies were conducted in animals using a model of acute full-thickness skin wounds and compared with Dexpanthenol, routinely used in clinical practice. The final part of our work involved an assessment of the histological findings and morphometry dynamics, with leukocyte and fibroblast count analysis of the wound tissues.

### 2.2. Synthesis of the Nanocomposites

#### 2.2.1. Collagen-Containing Solution

A commercially available product, a “Collagen-Containing Extract” (CCE) (Spes. No 9389-008-54969743-2016, JSC BIOMIR service, Moscow, Russia) was used as the collagen-containing matrix for cerium dioxide nanoparticles. This CCE serves as a base material for creating multicomponent homogeneous and microheterogeneous collagen-containing hydrogel scaffolds designed for use in bioengineered cellular products aimed at stimulating the regeneration of damaged tissues and organs [60,61,62]. CCE was obtained by acetic acid extraction from animal-derived tissues using patented technology [63]. The protein concentration in the CCE was 40 mg/mL (total protein content by dry weight: 96%), with a pH of 5.8 ± 0.3. The sterilizing dose required to achieve the established level of sterility was 15 kGy with an initial bioburden of hydrogel no more than 1.5 CFU (colony-forming units). After gamma sterilization the bioload was 0.0 CFU. Endotoxin level (LAL test) did not exceed 0.5 EU/mL. According to the manufacturers (and accompanying documents), the shelf life stability of the samples is 2 years. When the hydrogel was stored for at least two years, no changes in pH, sterility, or cytotoxicity were detected.

Subsequently, solutions with different collagen concentrations were prepared from the CCE by dilution in Dulbecco’s phosphate-buffered saline (PBS), pH 7.0–7.4, without Ca^2+^ and Mg^2+^ (PanEco Research and Production Enterprise, LLC, Moscow, Russia). The solutions were brought to the target concentrations under continuous stirring on a magnetic stirrer for 2 h at temperatures up to 45 °C. Each solution was prepared separately.

The investigated CCE concentrations were as follows: the stock CCE solution (in the article referred to as 100% collagen, viscous, undiluted); 50% collagen (1 part CCE + 1 part phosphate buffer); 25% (1 part CCE + 3 parts phosphate buffer); 20% (1 part CCE + 4 parts phosphate buffer); 16.7% (1 part CCE + 5 parts phosphate buffer); and 10% collagen (1 part CCE + 9 parts phosphate buffer). The obtained results, which indicated improved outcomes at low collagen concentrations, prompted us to include an additional group with 1% collagen in the study.

#### 2.2.2. Nanocerium

Two types of nanocerium, differing in their outer coatings (citric acid and polysaccharide dextran), were used in this study. To create nanocerium-citrate (CeO-CA) and nanocerium-dextran (CeO-Dex) complexes, Ce(NO_3_)_3_ × 6H_2_O (99.99%, LANHIT, Moscow, Russia; molecular weight 434.23 (326.13 anhydrous)) was used as the starting reagent in both cases.

The CeO-CA used in the study was synthesized according to a method detailed in our previous works, using initial Ce(NO_3_)_3_ × 6H_2_O and citric acid monohydrate (Sigma-Aldrich, C7129-100G, Batch, St. Louis, MO, USA) in a 1:1 molar ratio, as thoroughly described in our previous work [57,58]. CeO-Dex was synthesized by mixing Ce(NO_3_)_3_ × 6H_2_O and Dextran (ABCR GmbH, Karlsruhe, Germany) in a 1:2 ratio, as thoroughly described in our previous work [59]. According to the transmission electron microscopy data, the nanocerium particles had a size of less than 5 nm in the inorganic CeO_2_ core [57,58,59]. Nanocerium dilutions were prepared using sterile, bidistilled water.

**Figure 1:**
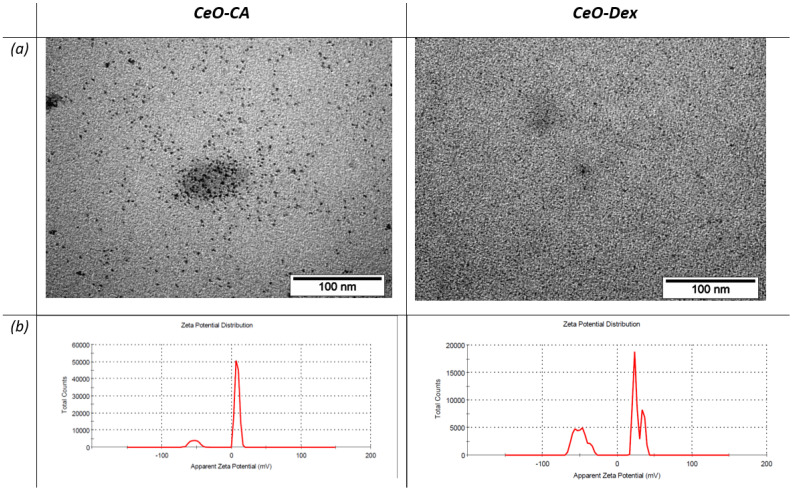
The results of analysis of nanoceria sols used to create hydrogels via transmission electron microscopy (**a**); electrokinetic zeta potential of sols after ultrasound treatment (**b**). On the left is CeO-CA NPS, and on the right is CeO-Dex NPs.

To create hydrogels, nanoparticles were synthesized using previously published methods, and the novelty of this work lies in the creation and evaluation of a new type of nanomedicines (nanocomposites). Therefore, in this article, we only present the main physical characteristics of these nanoparticles (Figure 1): the results of transmission electron microscopy that confirm size (<5 nm) and morphology (using JEOL 1200 TEM; JEOL, Tokyo, Japan), and diagrams of the electrokinetic zeta potential of the sols (using Malvern Zetasizer Nano ZS (Malvern Instruments Ltd., Worcestershire, UK), and Zetasizer Software v.2.3). In addition to the synthesis technology, the publications [57,58,59] also present more comprehensive results of physical and chemical studies of nanoparticles.

#### 2.2.3. Collagen–Nanocerium Composites

Mixing nanocerium sol and collagen resulted in the synthesis of collagen–nanocerium composites. First, specified concentrations of collagen were prepared. The nanocerium sol (CeO-Dex or CeO-CA) was added dropwise to the prepared collagen solution while stirring for 1 h on a magnetic stirrer for 1 h. Table 1 presents the final concentrations, nanocomposite components, and individual bases.

The physicochemical properties of the selected collagen–nanocerium composites (from the in vitro studies) and inclusion analysis were verified using FTIR spectroscopy. The study was performed using a Bruker VERTEX 70 FTIR spectrometer (Bruker Optik GmbH, Ettlingen, Germany) with a PIKE MIRacle ATR accessory for ATR, featuring a diamond crystal on zinc selenide. The following parameters were used for spectrum recording: number of scans, 32; spectral resolution—4 cm^−1^; spectral range—4000–600 cm^−1^; and the background spectrum was recorded immediately before each sample measurement.

### 2.3. Influence of Various Concentrations of Collagen–Nanocerium Composites and Their Components on Human Fibroblast Cytotoxicity and Proliferative and Metabolic Activity

The effects of nanocomposites and their components at various concentrations on the proliferative and metabolic activity of human fibroblasts, as well as their potential cytotoxic effects, were assessed in the BJ hTERT cell line. The human fibroblast cell line BJ hTERT was sourced from the American Type Culture Collection ATCC (Manassas, VA, USA), source—foreskin of a newborn. The cells belong to the hTERT-immortalized type obtained by transfecting the BJ fibroblast cell line with a plasmid expressing telomerase reverse transcriptase (hTERT). The assessment was performed using the MTT test and automated cell counting, with an assessment of the percentage of dead cells after 72 h of co-incubation with the test samples. The study was conducted according to the methodology detailed in our previous works [58,59,64].

Cell cultures were maintained in a CO_2_ incubator (Binder, Tuttlingen, Germany) under standard controlled conditions (5% CO_2_, 37 °C) and controlled humidity. The BJhTERT cell line was passaged every 7 days until the fibroblasts reached 100% confluency, following a standard protocol; the culture medium was changed every 3 days. The experiment was initiated after the cells reached 100% confluency. Cells were seeded in 24-well plates (SPL Life Sciences, Pocheon-si, Korea) using DMEM medium (PanEco Research and Production Enterprise, LLC, Moscow, Russia) according to a standard protocol at a cell suspension concentration of 5.0 × 10^4^/mL. The concentration was determined using an automated cell counter (Countess II Automated Cell Counter, Thermo Scientific, Waltham, MA, USA). The test samples were added to the wells at a volume of 10% of the total culture medium volume 24 h after passaging, followed by co-incubation in the CO_2_ incubator for the next 72 h. The control substances were added to the same volume.

Cell studies were conducted with results evaluated in the following groups to select the optimal collagen concentration: collagen concentrations of 100%, 50%, 25%, 20%, 16.7%, and 10%. The results were compared with those of the control group (culture medium with cells only) and the phosphate-buffered saline (PBS) group, which was used to prepare different collagen concentrations.

Our previous studies demonstrated that the maximum stimulation of cell metabolism and proliferation (including fibroblasts), with no sign of cytotoxicity, was observed for nanocerium samples at concentrations of 10^−3^ M to 10^−4^ M, regardless of the synthesis method or coating type [57,58,59,64]. Consequently, we investigated eight groups of collagen–nanocerium composite samples to identify the optimal variants (Table 2): CeO_2_ (10^−3^ M)-CA + Collagen (10%), CeO_2_ (10^−4^ M)-CA + Collagen (10%), CeO_2_ (10^−3^ M)-CA + Collagen (1%), CeO_2_ (10^−4^ M)-CA + Collagen (1%); CeO_2_ (10^−3^ M)-Dex + Collagen (10%), CeO_2_ (10^−4^ M)-Dex + Colagen (10%), CeO_2_ (10^−3^ M)-Dex + Collagen (1%), and CeO_2_ (10^−4^ M)-Dex + Collagen (1%). Two collagen groups (1% and 10%) were also investigated. The results were compared between the control and PBS groups.

#### 2.3.1. MTT Assay

After 72 h of incubation, the culture medium was removed and replaced with a 3% MTT reagent (PanEco Research and Production Enterprise, LLC, Moscow, Russia), prepared from a 5 mg/mL stock solution, and incubated at 37 °C. Subsequently, the working MTT solution was aspirated, and dimethyl sulfoxide (DMSO; PanReac AppliChem, Darmstadt, Germany) was added. The plate was then placed on an Elmi-S4 rocking platform (ELMI, Riga, Latvia) at room temperature for 5 min to achieve homogeneity in the wells. Optical density (OD) was measured using a Multiscan Labsystems spectrophotometer (Multiscan Labsystems, Vantaa, Finland) at a wavelength of 540 nm. The final results are expressed as relative OD units. Each sample (from each group) was tested in at least 12 replicates.

#### 2.3.2. Quantitative Cell Counting and Cytotoxicity Assessment

Quantitative cell counting with cytotoxicity assessment, based on evaluating cell membrane permeability using the vital dye Trypan Blue, was performed using a Countess II Automated Cell Counter (Thermo Scientific, Waltham, MA, USA), according to a previously described methodology [58,59,64]. After 72 h of co-incubation, the culture medium containing the test compounds was removed from the plate, and the wells were washed with phosphate buffer (pH 7.4) (PanEco Research and Production Enterprise, LLC, Moscow, Russia). Gentle cell detachment was achieved by adding a 4:1 Versene/Trypsin solution (PanEco Research and Production Enterprise, LLC, Moscow, Russia) in a 4:1 ratio and incubating in a thermostat for 1 min. Detached fibroblasts were collected in an Eppendorf tube containing phosphate buffer collected from the washed wells to preserve the cells. The samples were thoroughly pipetted, followed by the addition of 0.4% Trypan Blue solution (PanEco Research and Production Enterprise LLC, Moscow, Russia). Direct cell counting was performed using disposable counting slides. The total cell concentration per unit volume was expressed as the number of cells (×10 cells), and the percentage of live and dead cells was calculated.

### 2.4. Bacterial Biosensors

The selected nanocomposite prototypes (CeO-CA + Collagen and CeO-Dex + Collagen) were studied using bacterial biosensors to determine their biological activity and safety. Four types of bioluminescent *E. coli* were used in this study (Table 3). This allowed us to study the following parameters: general toxicity, antioxidant and pro-oxidant activities, and antigenotoxic and promutagenic activities.

Whole-cell biosensors were chosen for this study because they are capable of detecting only bioavailable compounds (biologically active parts) that can be absorbed by the cell and interact with its components [65,66].

The biosensor strain *E. coli* MG1655, which carries a recombinant plasmid with the lux operon from the luminescent bacterium *P. luminescens* fused to inducible gene promoters, is used to assess toxicity and identify potential genotoxicants and antioxidants among chemical compounds [67,68,69].

The *E. coli* MG1655 pXen7 strain carries a constitutive, constantly expressed lac promoter, and is used to detect general toxicity or the effect of test substances on the bioluminescence process [70]. This allows the exclusion of false-positive results when testing inducible biosensors on the same line [71,72]. The collagen–nanocerium composites were tested on the *E. coli* MG 1655 pXen7 biosensor for general toxicity, recorded as luminescence suppression relative to the control. Luminescence was assessed using absolute values and relative units (percentage of the control).

Inducers were used to study the other three biosensor strains using the following parameters: luminescence level (before/after induction), induction coefficient (IC), and protective (antioxidant, antimutagenic) effects.

The SOX-inducible promoter PrecA of the *E. coli MG1655 pRecA strain* detects the penetration of DNA-damaging agents into cells [65,73]. The bioluminescent biosensor *E. coli* MG1655 pRecA was used to study the promutagenic and antigenotoxic activities of the collagen–nanocerium composites. This strain has been used to test substances for their ability to damage and protect DNA [74,75,76,77].

The *E. coli* MG1655 pKatG and *E. coli* MG1655 pSoxS strains, with promoters inducible by peroxides and superoxide, respectively, detect oxidative stress [66]. These strains were used to assess the redox activity of the nanocomposites. The pro-oxidant activity was assessed based on the ability of the test samples to induce stress-inducible promoters (enhanced luminescence). Antioxidant activity was assessed based on the ability of the samples to reduce the induction caused by the test inducer. The effects were tested in two models: against paraquat, a generator of reactive oxygen species (ROS), the superoxide anion radical (SAR) (*E. coli* MG1655 pSoxS), and hydrogen peroxide, H_2_O_2_ (*E. coli* MG1655 pKatG).

#### Bioluminescent Test with Inducible *E. coli* Strains

The bioluminescent *E. coli* MG1655 strain was cultured in LB medium containing ampicillin (125 µg/mL). The microbial suspension was diluted to a density of 0.05 McFarland units 30 min before the main experiment.

The culture turbidity was measured using a DEN-1B densitometer (Biosan, Rīga, Latvia). Subsequently, 160 µL aliquots of this culture were transferred to sterile wells of a Greiner 96 Flat-Bottom microplate (Greiner Bio-One, Kremsmünster, Austria). Next, 20 µL of the test substance (nanocomposite) was added to each well. The wells with the addition of solvents–20 µL of distilled water and 20 µL of PBS buffer (solvent control)–were used as controls. The plate was then incubated for 15 min in a thermostat at 37 ± 0.2 °C, and luminescence was measured.

Subsequently, 20 µL of inducer was added to a 96-well plate. For testing antioxidant activity, hydrogen peroxide H_2_O_2_ (JSC Ekos-1, Moscow, Russian) was added at a final concentration of 400 µM in the well, and the SAR generator Paraquat (Methyl Viologen Hydrate 98%, C_12_H_14_Cl_2_N_2_ × H_2_O crystalline powder, molecular weight 257.15, Thermo Fisher Scientific, Dreieich, Germany) was added at a final concentration of 400 µM. To assess genotoxic stress, Dioxidine (1,4-dioxide of 2,3-quinoxalinedimethanol) (PJSC Biosintez, Penza, Russia) was added at a final concentration of 9 µM [66].

The plate was placed in a FLUOstar Omega multimodal reader version 5.10 (BMG LABTECH, Ortenberg, Germany) with a thermostat and incubated at 37 °C, 200 rpm (double orbital) to assess luminescence intensity.

Bioluminescence intensity was measured every 10 min for 120 min.

The final luminescence result (Lum) was expressed in relative light units (RLU).

The IC was calculated using the following formula:

(1) $$IC=\frac{L_{e}}{L_{c}}-1$$

where

*L_e_* is the luminescence intensity of the sample with an inducer (in arbitrary units).

*L_c_* is the control sample’s luminescence intensity (in arbitrary units).

The protective effect (*P*) was calculated using the following formula:

(2) $$P=\left(1-\frac{I_{e}}{I_{c}}\right)\times 100\%$$

where

*I_e_* and *I_c_* are the luminescence ICs in the presence and absence of the nanoparticle solutions, respectively. Particular attention was paid to the maximal protective effect.

### 2.5. In Vivo Studies of Collagen–Nanocerium Hydrogel Drug Prototypes to Determine Efficacy in an Animal Model of Acute Full-Thickness Skin Wound

The efficacy of collagen–nanocerium hydrogel composites (final concentrations of 0.001 M CeO_2_ and 10% CCE) was studied in 40 male Wistar rats weighing 200–255 g using a model of acute full-thickness skin wounds. The rats were obtained from the breeding nursery of the Federal State Budgetary Institution of Science “Scientific Center for Biological Technologies of the Federal Medical-Biological Agency,” Branch “Stolbovaya.” The animals were kept under standard vivarium conditions with a 12 h light/12 h dark cycle and free access to water and food (30 g of feed per day per animal). After wound modeling, the rats were kept individually in cages to prevent mutual trauma and licking of the preparations by other animals.

Full-thickness skin wound modeling was performed under general isoflurane anesthesia (induction in a chamber with 3% mixture enrichment at a flow rate of 1 L/min, maintenance using a mask with 2.5% mixture enrichment at a flow rate of 0.8 L/min). The surgical field was pre-prepared by shaving the hair and treating it with 70% ethyl alcohol. Two standardized wounds (square wound size: 10 × 10 mm; depth: down to the fascia) were modeled on the back of each rat at equal distances to the right and left of the spine. A patented device was used to model wound surfaces of specified sizes in laboratory animals, according to the method detailed in our previous studies [14,78,79,80]. A total of 80 wounds were simulated, with 20 wounds allocated to each experimental group. An experimental unit was defined as a single wound on an individual animal. The number of animals was progressively reduced during the study through euthanasia at predefined time points.

#### 2.5.1. Treatment and Groups

The investigated nanodrug prototypes were applied to the wound or wound area once daily at a dose of 0.2 mL until complete wound healing and the formation of a connective tissue scar, regardless of changes in wound size. Four experimental wound groups were analyzed, which differed in their treatment methods. Two study groups were investigated: CeO-CA + Collagen and CeO-Dex + Collagen groups. Dexpanthenol (5% ointment for external use) was selected for comparison. This medication belongs to the pharmacological group of regenerative drugs and is indicated for the treatment of wounds and burns (Gross formula: C_9_H_19_NO_4_, Tatkhimpharmpreparaty, Kazan, Russia). The control group consisted of wounds treated with a 0.9% NaCl solution (0.2 mL).

Group randomization was based on the weight measured before and after the quarantine period. All animals were of the same sex and age, and were from the same batch at the kennel.

#### 2.5.2. Assessment of Effectiveness

The reduction in wound surface area was the primary clinical efficacy criterion.

To assess wound size dynamics on days 1, 3, 7, and 14 of the experiment, macro-photography of the animal wounds was performed from a fixed distance using a tripod and a Canon EOS550D digital camera with a Canon EF-S18-55 lens (focal length 50 mm, distance to subject 30 cm). A ruler with millimeter divisions was positioned within the frame, adjacent to the wound. Each image was analyzed using JMicroVision 1.2.7.

The animal sacrifice time points were days 3, 7, and 14, with tissue collection for subsequent histomorphometric analysis. Skin fragments containing wounds were collected from rats after euthanasia (exsanguination via right ventricle puncture under general anesthesia using chloral hydrate at 300 mg/kg).

To comply with the commonly accepted “3Rs” [81] principles, in particular “Reduction in number of animals used”, it was decided, with the approval of the ethics committee, that the minimum number of study units (wounds) at each withdrawal point for each group was 5. If an animal died earlier, an additional animal would take its place. However, there were no exceptions or conclusions from the study.

#### 2.5.3. Histological and Morphometric Examination of the Wound Tissues

The experimental wound area was excised along with the surrounding intact skin, down to the muscles of the back. The wound tissues were then numbered and transmitted to the histologists in encrypted form. Thus, the histomorphologists were blinded.

The obtained tissue was fixed in a buffered 10% neutral formalin solution for 14 days. After standard tissue processing, embedding in paraffin, and mounting tissue blocks on cassettes, serial sections 5 µm thick were prepared using a CUT 5062 rotary microtome (SLEE Medical Gmbh, Nieder-Olm, Germany). Sections were stained with hematoxylin and eosin for classical histology and microscopy, and fibroblastic and leukocytic cells were assessed using Leica Autostainer XL (ST5020) (Leica Biosystems, Deer Park, IL, USA). Light microscopy examination was performed using a Nikon Eclipse Ni-U microscope with an imaging and morphometry workstation (Nikon, Tokyo, Japan) and a Hamamatsu NanoZoomer-SQ histology slide scanner (Hamamatsu Photonics, Hamamatsu city, Japan) at 40× objective magnification. The Hamamatsu NanoZoomer SQ software system (Hamamatsu Photonics, Hamamatsu city, Japan) was used to perform the linear measurements. Microimages of individual granulation tissue layers were taken, and the corresponding cells (leukocytes and fibroblasts) were counted using the ImageJ 1.54h software.

#### 2.5.4. Ethics

Animal experiments were conducted in accordance with the principles of handling laboratory animals and provisions of the European Convention for the Protection of Vertebrate Animals Used for Experimental and Other Scientific Purposes (CETS 123).

This study was approved by the two ethics committees. The study was approved by the Regional Ethics Committee of the Federal State Budget Educational Institution of Higher Education at the Kursk State Medical University of the Ministry of Health of Russia (Protocol No. 3 dated 13 September 2023) and the Local Ethics Committee of Sechenov University (protocol No. 19–23 dated 26 October 2023).

### 2.6. Statistical Analysis

Statistical analysis was performed using the SPSS 25.0 statistical program (IBM Corp., Armonk, NY, USA). The normality of the distributions of MTT test parameters and cell counts for each sample was assessed using the Kolmogorov–Smirnov and Shapiro–Wilk criteria.

#### 2.6.1. Cultural Studies on Fibroblasts

Each sample (each group) was tested in at least 12 assays in MTT (т = 12); the sample size (n) in each group for quantitative cell counting was from 7 to 10. The normality test confirmed obedience to the law of normal distribution (*p* > 0.05). One-factor analysis of variance (ANOVA) was performed for the comparative analysis of different subgroups. Multiple posterior comparisons were performed using Dunnett’s test (for comparison with controls). Differences were considered statistically significant when the *p*-value was <0.05.

#### 2.6.2. Lux-Biosensor Study

Each sample of each concentration in each lux-biosensor test was tested at least 3 times. Statistical significance was determined using Student’s *t*-test for independent samples at *p* < 0.05.

#### 2.6.3. Statistical Analysis of Animal Study Results

To analyze the size of wounds, the sample size varied and was maximum on day 1 of the study (n = 20 in each group), and then decreased as animals were withdrawn from the experiment. The sample size for analyzing wound tissue at each study site (including the number of leukocytes and fibroblasts in the wound tissue histology) was 5 (n = 5). In general, the samples did not follow a normal distribution; therefore, the Kruskal–Wallis test with the Bonferroni correction was used for multiple comparisons of independent samples. Differences were considered statistically significant when the *p*-value was <0.05.

## 3. Results

### 3.1. FTIR Spectroscopy Results of Nanocomposites

**Figure 2:**
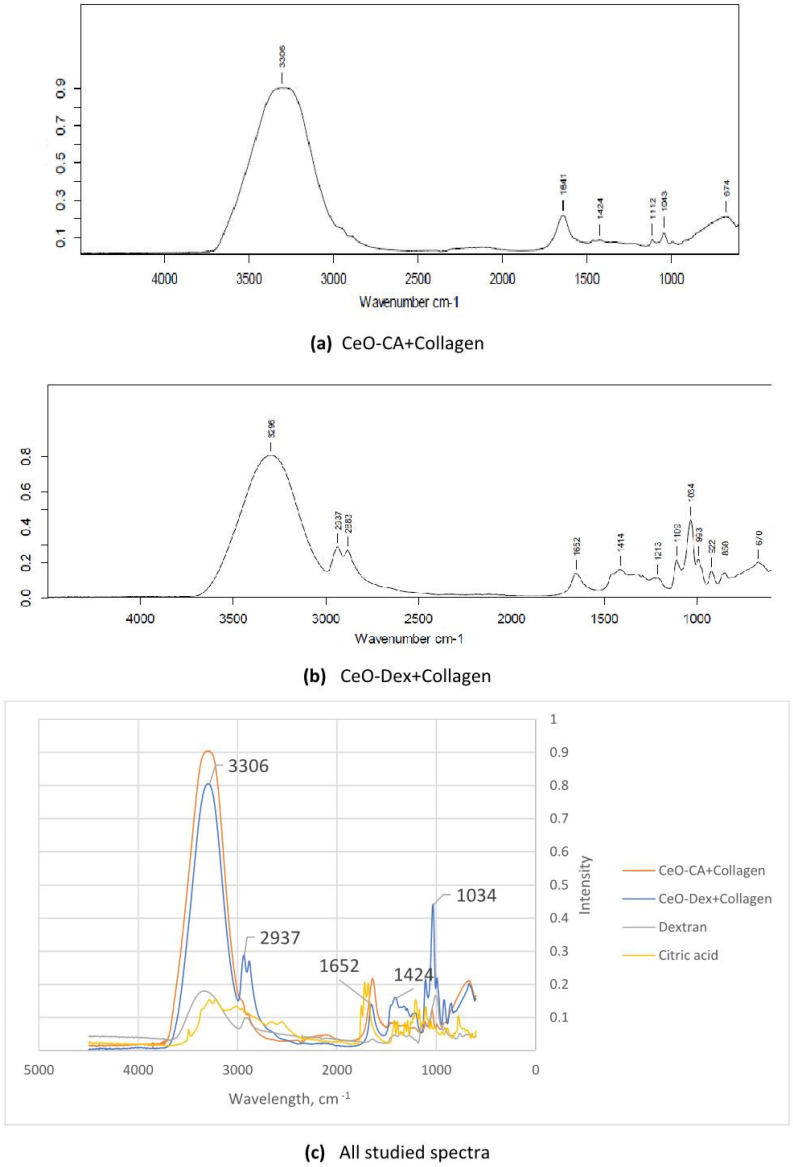
FTIR spectroscopy of the (**a**) CeO-CA + Collagen sample, (**b**) CeO-Dex + Collagen sample, and (**c**) CeO-Dex + Collagen (blue), CeO-CA + Collagen (orange), citric acid (yellow), dextran (gray).

Investigation of CeO-CA + Collagen revealed the following absorption bands for the composite material: ν (H_2_O) = 3306 cm^−1^ (str.), ν_as_ (COO^−^) = 1641 cm^−1^ (m.), ν (C-C) = 1112 cm^−1^ (w.), ν_as_ (P-O), ν (Ce-O) = 1043 cm^−1^ (w.), δ (P-O) = 674 cm^−1^ (m.). A shift in the main signals of citric acid for the valence skeletal vibrations (C-C-) was noted, which can be attributed to the influence of newly formed bonds in the composite material (Figure 2a,c).

Investigation of CeO-Dex + Collagen revealed the following absorption bands for the composite material: ν (H_2_O) = 3296 cm^−1^ (str.), ν (C-H) = 2937 cm^−1^, 2883 cm^−1^ (m.), ν_as_ (COO^−^) = 1652 cm^−1^ (m.), ν (C-C) = 1109 cm^−1^ (w.), ν_as_ (P-O), ν (Ce-O), ν (C-O-C) = 1043 cm^−1^ (m.), δ (O-H) = 674 cm^−1^ (w.). A shift in the main signals of dextran for the valence skeletal vibrations -C-C-, vibrations of the C-O-C grouping of glycosidic bonds, and specific vibrations of the pyranose ring of glucose were observed, along with the appearance of new absorption bands corresponding to the deformation vibrations of C-O-C bonds in esters, deformation vibrations of C-O bonds in rings or cyclic structures, and deformation skeletal vibrations of -C-C-. This can be attributed to the influence of the newly formed bonds in the composite material (Figure 2b,c).

Thus, the FTIR spectroscopy results confirmed the formation of new bonds, that is, nanocomposites capable of possessing novel pharmacological properties.

### 3.2. Results of the Cell Culture Studies

#### 3.2.1. Optimal Concentrations of Composite Components Stimulating Human Fibroblast Activity

Our previous studies demonstrated that the maximum stimulation of cell metabolism and proliferation (including fibroblasts and keratinocytes) in the absence of cytotoxicity was observed at nanocerium concentrations of 10^−3^ M to 10^−4^ M, regardless of the synthesis method or coating type [58,59,64]. This stage aimed to select the optimal concentration of the collagen biopolymer that best stimulated the key cells involved in skin wound regeneration (fibroblasts) for the subsequent development of an effective wound healing drug.

**Figure 3:**
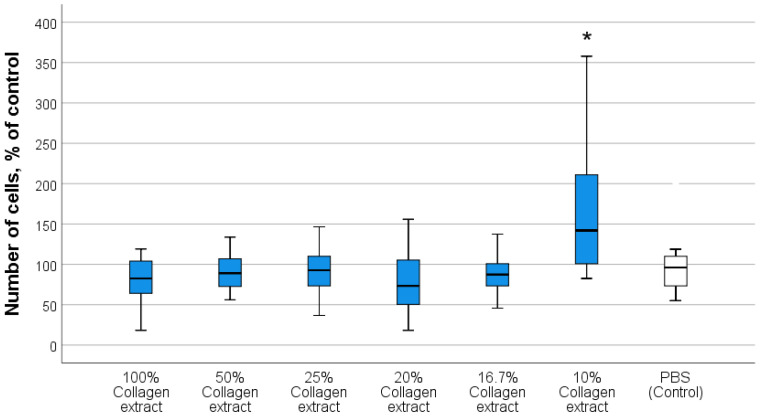
Effect of different collagen concentrations on the proliferative activity of BJ hTERT cells (cell counter) (ANOVA: F = 4.05, *p* = 0.02; *—difference from control, Dunnett’s test). The white boxplot represents the control against which the study groups were compared.

Results from the direct quantitative counting of fibroblasts indicated the superiority of 10% collagen extract. Analysis of variance revealed statistically significant differences compared with the PBS control group (on average 1.72-fold higher) and between all investigated collagen groups in the concentration range of 16.7–100% (*p* < 0.05). Other collagen concentrations showed no difference from the control in terms of fibroblast cell number after 72 h of co-incubation (Figure 3).

**Figure 4:**
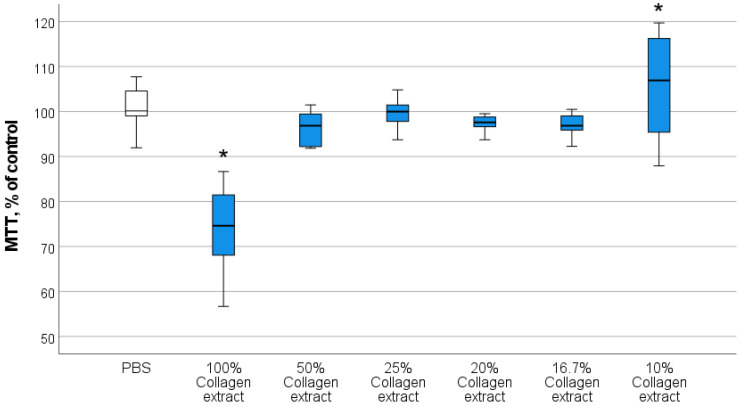
Effect of different collagen concentrations on the metabolic activity of the BJ hTERT cell line in the MTT test (ANOVA OD: F = 51.49; *p* < 0.001; *—*p* < 0.001 compared with the control according to Dunnett’s test). The white boxplot represents the control against which the study groups were compared.

Analysis of the MTT test results using ANOVA revealed a statistically significant difference in OD values between the groups included in the experimental design. A stimulating effect on the metabolism of human fibroblasts was recorded for 10% collagen extract (106 ± 10.7% relative to the control; *p* < 0.05). According to the MTT assay, the metabolism of fibroblasts after 72 h of co-incubation with 100% collagen extract alone was statistically lower than that in the control (on average 74% relative to the control, *p* < 0.01) and compared with all other groups. This is likely due to the thickness of the coating on the cell culture at high collagen concentration and the associated high viscosity of the hydrogel, which impeded the respiratory metabolism of fibroblasts. Other concentrations of collagen extract (50%, 25%, 20%, and 16.7%) had no significant effects on the MTT test results, averaging 96–100% relative to the control, with no statistically significant differences (Figure 4).

When assessing the live-to-dead cell ratio using Trypan Blue staining, no statistically significant differences were found between the experimental groups of 10–50% collagen. Dead cells were mostly absent, or their percentage was minimal (up to 6% per well). This demonstrated the absence of cytotoxicity, that is, the safety of the collagen extract.

The obtained data indicated the safety and absence of cytotoxicity of the collagen extract, the stimulating effect of the 10% collagen extract on the proliferative and metabolic activity of human fibroblasts, and the lack of an effect on fibroblast number at higher collagen concentrations. The latter is associated with the fact that an excess of collagen, and consequently amino acids (which fibroblasts use to synthesize collagen), means that fibroblasts do not require active metabolism and enter a phase of metabolic quiescence.

Thus, the stimulating effects of the 10% collagen extract concentration justified the preference for selecting a final collagen extract concentration not exceeding 10% (4 mg/mL protein) for preparing the nanocomposite.

These results prompted us to investigate not only 10%, but also 1% collagen for creating nanocomposites, despite the nanocomposite viscosity level with 1% collagen extract not meeting our requirements.

#### 3.2.2. Investigation of the Effect of Collagen–Nanocerium Composites on Proliferative and Metabolic Cell Activity

The next step in our research was to evaluate the effect of the nanocomposites on human fibroblasts. We used two types of nanocerium (CeO-CA, CeO-Dex) at two concentrations (10^−3^ M and 10^−4^ M) in combination with two concentrations of collagen extract (10% and 1%). Eight groups of collagen–nanocerium composite drug prototypes were studied.

The study found that all investigated nanocomposites, as well as the 1% and 10% collagen used to create them, differed significantly from both the control group (*p* < 0.001) and the PBS group used for collagen dilution (*p* < 0.001). Collagen concentrations of 1% and 10% increased the fibroblast metabolism indicator by an average of 16–17% (*p* < 0.01), with no difference in the effect between the 1% and 10% collagen concentrations.

The addition of nanocerium at a concentration of 10^−4^ M to collagen led to an enhancement of human fibroblast metabolism by an average factor of 1.27–1.31 for CeO-Dex and 1.30–1.31 for CeO-CA (*p* < 0.01). This indicates that cerium dioxide nanoparticles at a concentration of 10^−4^ M in complex with 1–10% collagen exert an identical stimulating effect on cell metabolism (increasing it by an average of 30%), regardless of the nanocerium synthesis method and collagen concentration used in the nanocomposite. The absence of a collagen dose-dependent effect indicates that the mere presence of collagen, even at small concentrations, may serve as a trigger for cells to actively grow and differentiate.

**Figure 5:**
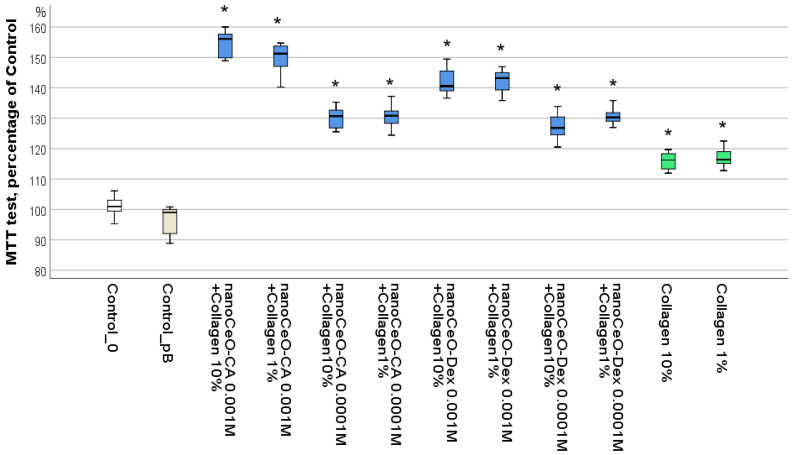
Stimulation of human fibroblasts during co-incubation with various nanoceria-collagen complexes in the MTT test, percentage of Control_0 (ANOVA OD: F = 291.9; df 11, *p* < 0.001; *—different from Control_0 at *p* < 0.001; Dunnett *t*-tests; n = 12 in each group, N = 144 in total). The white boxplots represents the control against which the study groups were compared; Blue boxplots represent the hydrogel nanocomposite groups under study; Green boxes represent the comparison groups containing only collagen.

The highest OD indicator level, reflecting fibroblast activation, was recorded during co-incubation with nanocomposites containing nanoceria at a concentration of 10^−3^ M. The concentration of the collagen extract in these cases did not affect the OD value. The maximum level of fibroblast metabolism was recorded for CeO-CA (10^−3^ M) + Collagen (the OD indicator was on average 55% higher than the control for co-incubation with CeO-CA + 10% collagen and 50% higher for co-incubation with CeO-CA + 1% collagen). Fibroblast metabolism relative to the control after co-incubation with CeO-Dex + Collagen averaged 142% of that of the control (*p* < 0.01). No statistically significant differences were recorded between the CeO-CA + Collagen and CeO-Dex + Collagen groups at a CeO_2_ nanoparticle concentration of 10^−3^ M in the final preparation, both in the composites with 1% and 10% collagen (Figure 5).

The lack of difference between the 1% and 10% collagen extracts prompted us to use 10% collagen in animal studies, justified by the higher viscosity of the nanocomposite, which, from our perspective, is more convenient for applying the regenerative drug to the skin wound surface.

Thus, the conducted cell culture studies allowed for the selection of the most active and safe collagen–nanocerium complex composites—CeO (10^−3^ M)-CA + Collagen (10%) and CeO (10^−3^ M)-Dex + Collagen (10%), which significantly stimulated fibroblasts by 1.5 and 1.4 times, respectively. These nanodrug prototypes were used in subsequent studies.

### 3.3. Results of the Bacterial Biosensor Studies

#### 3.3.1. Results of Collagen–Nanocerium Composites Testing for General Toxicity on the *E. coli* MG 1655 pXen7 Biosensor

**Figure 6:**
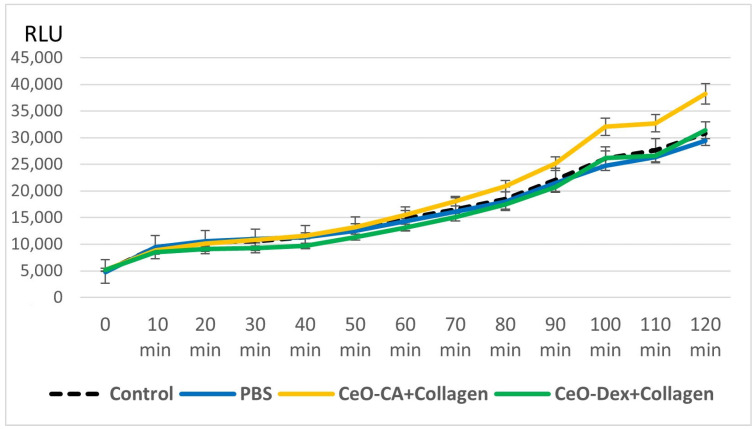
Bioluminescence of the *E. coli* MG 1655 pXen7 biosensor strain under the influence of collagen–nanocerium composites.

Throughout the study, bioluminescence of the *E. coli* MG 1655 pXen7 strain was recorded every 10 min during co-incubation with the nanocomposites, with levels generally corresponding to the control. However, several significant differences were observed. The CeO-Dex + Collagen composite demonstrated a statistically non-significant trend toward inhibition of bioluminescence by an average of 12–15% between the 20th and 60th min of the study (*p* > 0.05); after 60th minutes, this trend faded. Culture of *E. coli* MG 1655 pXen7 with the CeO-CA + Collagen complex revealed no inhibitory effect throughout the study, with no suppression of luminescence. On the contrary, an intensification of luminescence was recorded, averaging 18–24% relative to the control, starting from the 100th minute and continuing until the endpoint of the study (Figure 6).

Thus, the bioluminescence study on the lux-biosensor *E. coli* MG 1655 pXen7 confirmed the absence of toxicity of the collagen–nanocerium composite.

#### 3.3.2. Antigenotoxic and Promutagenic Activity of Collagen–Nanocerium Composites on the *E. coli* MG1655 pRecA Biosensor

**Figure 7:**
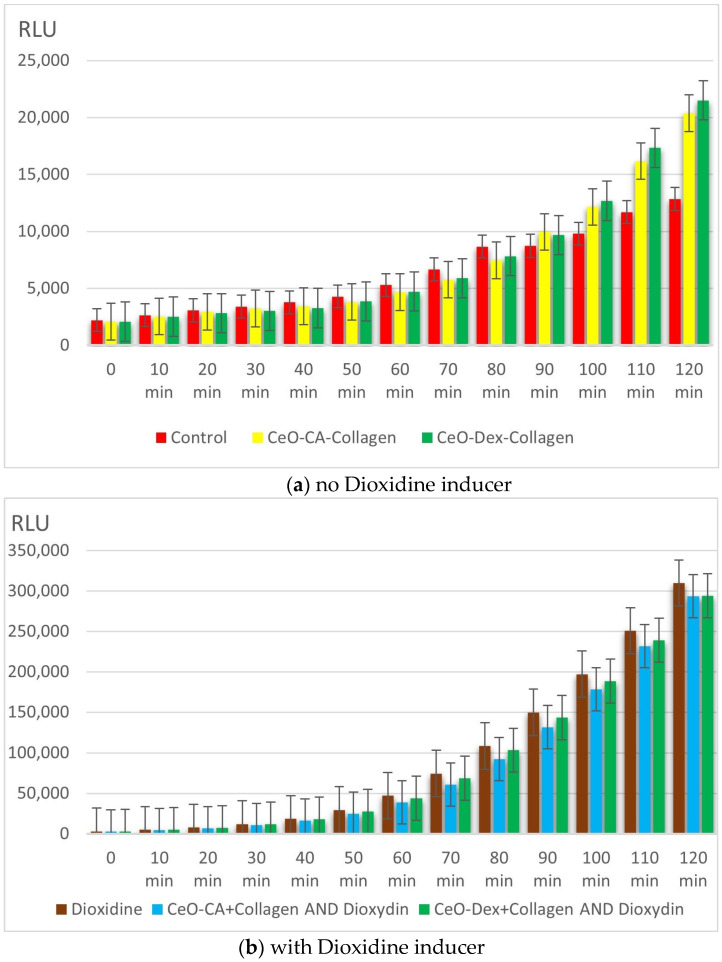

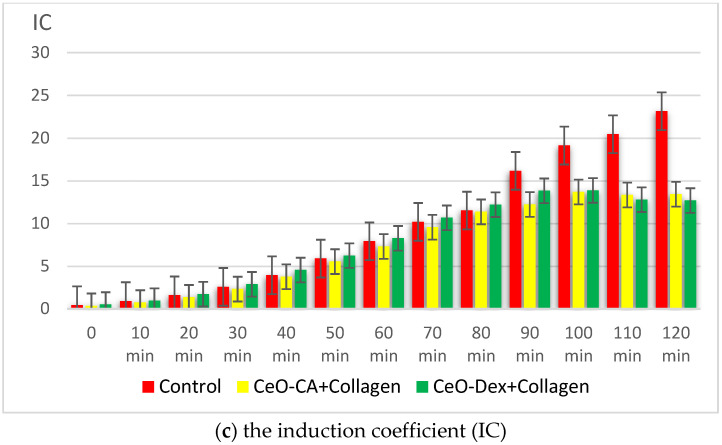
Bioluminescence of the *E. coli* MG1655 pRecA biosensor strain under the influence of collagen–nanocerium composite samples without and with the addition of an inducer.

No promutagenic effect associated with DNA damage was established for any collagen–nanocerium composite samples using the bioluminescent strain *E. coli* MG1655 pRecA (Figure 7).

Testing without adding a DNA damage inducer showed that at 100–120 min of measurement, the nanocomposites exerted a stimulating effect on the bacteria (on average 1.2–1.7 times), which can be interpreted as an improvement in the RecA protein-mediated DNA repair pathway. Upon addition of the DNA damage inducer Dioxidine (positive control), the luminescence of *E. coli* MG1655 pRecA-lux bacteria increased 23-fold by the 120th minute of the study. For the nanocomposite groups, the maximum IC was 13.7 for CeO-CA + Collagen and 13.9 for CeO-Dex + Collagen (maximum recorded at 100 min). Hence, the protective effect of the nanocomposites was 42% in the CeO-CA + Collagen group and 45% in the CeO-Dex + Collagen group (*p* < 0.05). A clear protective effect was recorded between 90 and 120 min, reaching its maximum level at the 120th minute of the study. No significant differences were observed between the nanocomposite groups (Figure 7).

Thus, the nanocomposites do not possess significant promutagenic activity but exhibit moderate antigenotoxic activity, protecting DNA from damage by up to 45%.

#### 3.3.3. Redox Activity of Collagen–Nanocerium Composites on the Bioluminescent Biosensors *E. coli* MG1655 pSoxS-lux and pKatG

**Figure 8:**
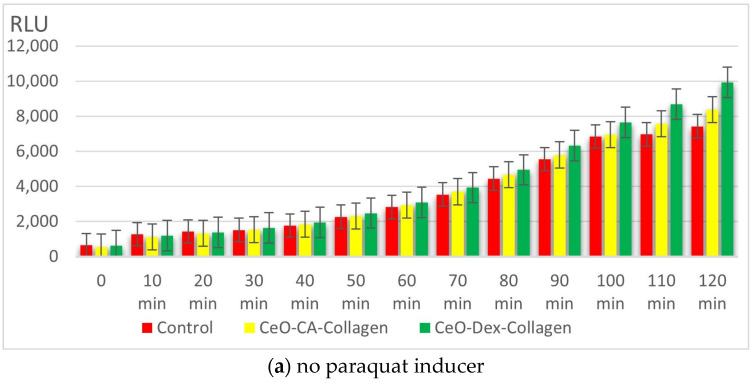

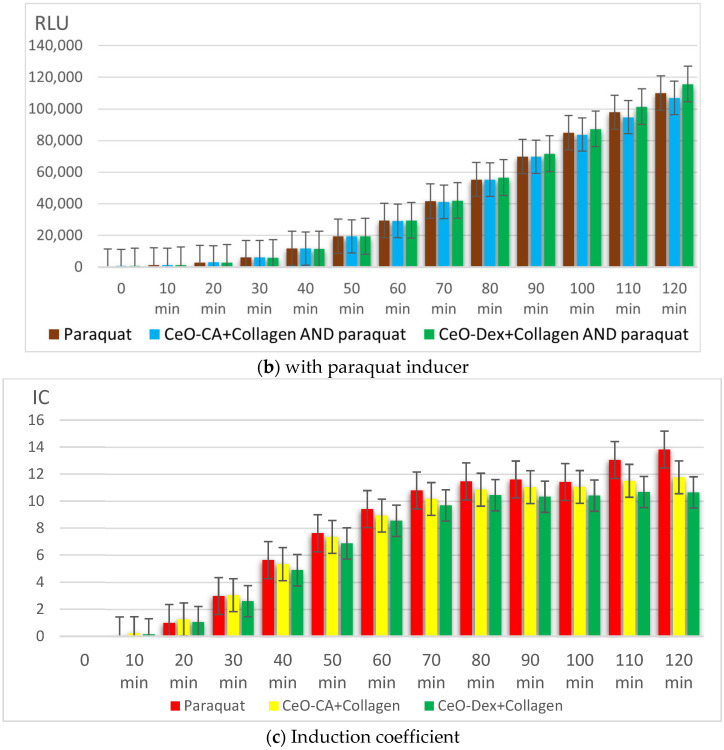
Dynamics of bioluminescence of the *E. coli* MG1655 pSoxS-lux strain under the influence of collagen–nanocerium composite samples with and without the addition of paraquat, an SAR formation inducer.

Testing with the lux-biosensor strain *E. coli* MG1655 pSoxS-lux, a sensor sensitive to paraquat, an intracellular inducer of the superoxide radical (SAR), showed that the studied nanocomposites lacked a pro-oxidant effect, which was demonstrated when oxidative stress was modeled by adding paraquat. Upon addition of paraquat, the IC increased dynamically, reaching a maximum at the 120th minute: 13.8 in the positive control, 11.8 in the CeO-CA + Collagen group, and 10.7 in the CeO-Dex + Collagen group. After adding paraquat, an antioxidant protective effect against ROS was recorded, most pronounced for the CeO-Dex + Collagen composite (averaging 9–23% between the 30th and 120th minutes of the study). The maximum protective effect in this group was recorded at the 120th minute, when the CeO-Dex + Collagen composite inhibited SAR formation by 23%. The CeO-CA + Collagen composite also possessed a protective effect, but it was 1.5 times weaker, averaging 3–15% with a maximum at the 120th minute of the study (Figure 8).

**Figure 9:**
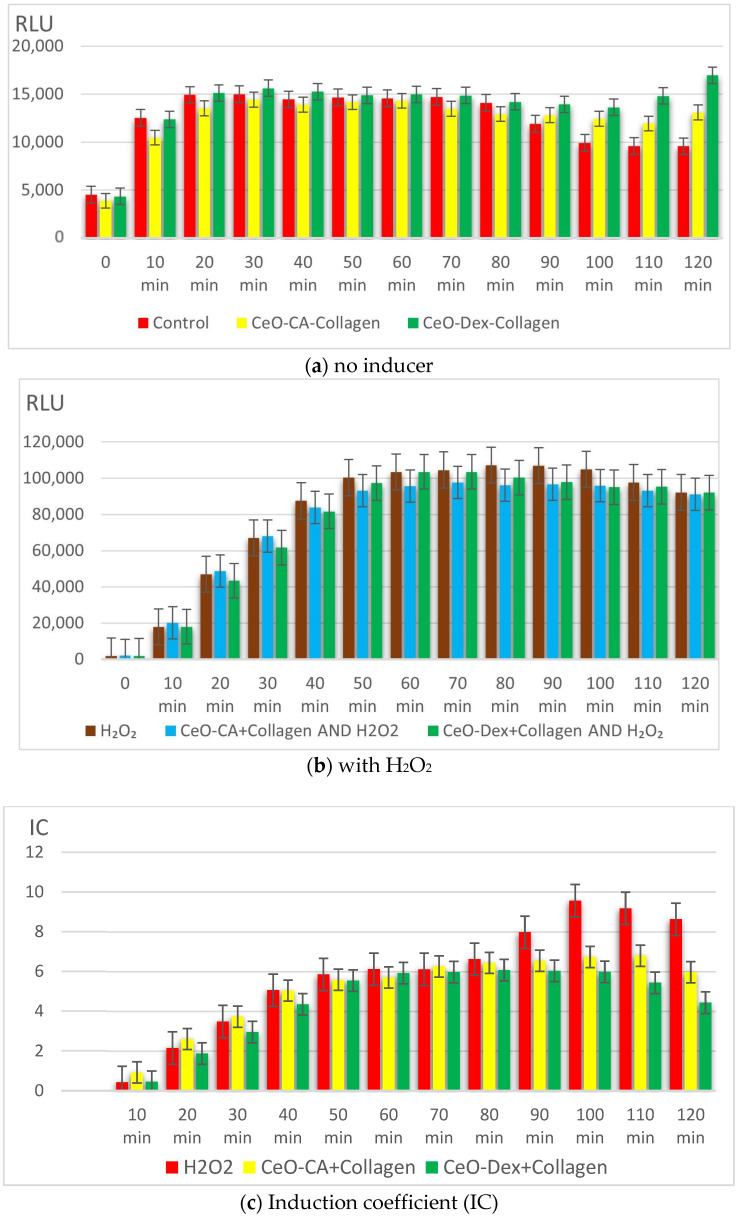
Dynamics of bioluminescence of the *E. coli* MG1655 pKatG strain under the influence of collagen–nanocerium composite samples with and without hydrogen peroxide addition.

Analysis of the bioluminescence dynamics of the *E. coli* MG1655 pKatG strain demonstrated an antioxidant effect against hydrogen peroxide, which is characteristic of CeO-Dex + Collagen. The addition of H_2_O_2_ significantly enhanced bacterial luminescence from the 20th minute onwards (IC > 2). The IC in the control increased to 8–9.6 during the 90–120 min period, with a maximum at the 100th minute of the study. The nanocomposites provided an antioxidant effect; in the CeO-CA + Collagen group, the IC increased to a maximum of 6.8 (at 100 min), and in the CeO-Dex + Collagen group—to 6.0 (at 90–100 min). The maximum protective antioxidant effect against H_2_O_2_ was 30.9% in the nanoCeO-CA + Collagen group and 48.7% in the CeO-Dex + Collagen group. No pro-oxidant properties were identified for the CeO-Dex + Collagen composite in the modeled oxidative stress reaction with added H_2_O_2_ (Figure 9).

Thus, a study on oxidative stress biosensors showed that collagen–nanocerium composites possess statistically significant antioxidant activity, most pronounced against hydrogen peroxide. The application of the CeO-Dex + Collagen composite inhibited SAR formation by up to 23% and CeO-CA + Collagen by up to 15%. The maximum antioxidant effects against peroxide were recorded at 49% and 31% in the CeO-Dex + Collagen and CeO-CA + Collagen groups, respectively. Therefore, CeO-Dex + Collagen exhibited the most pronounced antioxidant properties.

### 3.4. Efficacy of Drug Prototypes Based on Cerium Dioxide Nanoparticles and Collagen Biopolymer in an Animal Model of Acute Full-Thickness Skin Wound

#### 3.4.1. Wound-Healing Dynamics

By the end of the 1st day, the wound surface in all animal groups was covered with dense crust formed from dried exudate, clotted blood, and fibrin. In rats from groups treated with collagen–nanocerium composites and the comparison drug, hardened components of the medications also contributed to the formation of the wound crust (eschar). Therefore, in all experimental groups, the acute skin wounds healed under an eschar, and the wound surface area was assessed by the reduction in the size of the wound crust due to drying and partial resorption. No cases of wound suppuration related to open wound contamination were recorded during the experiment.

**Figure 10:**
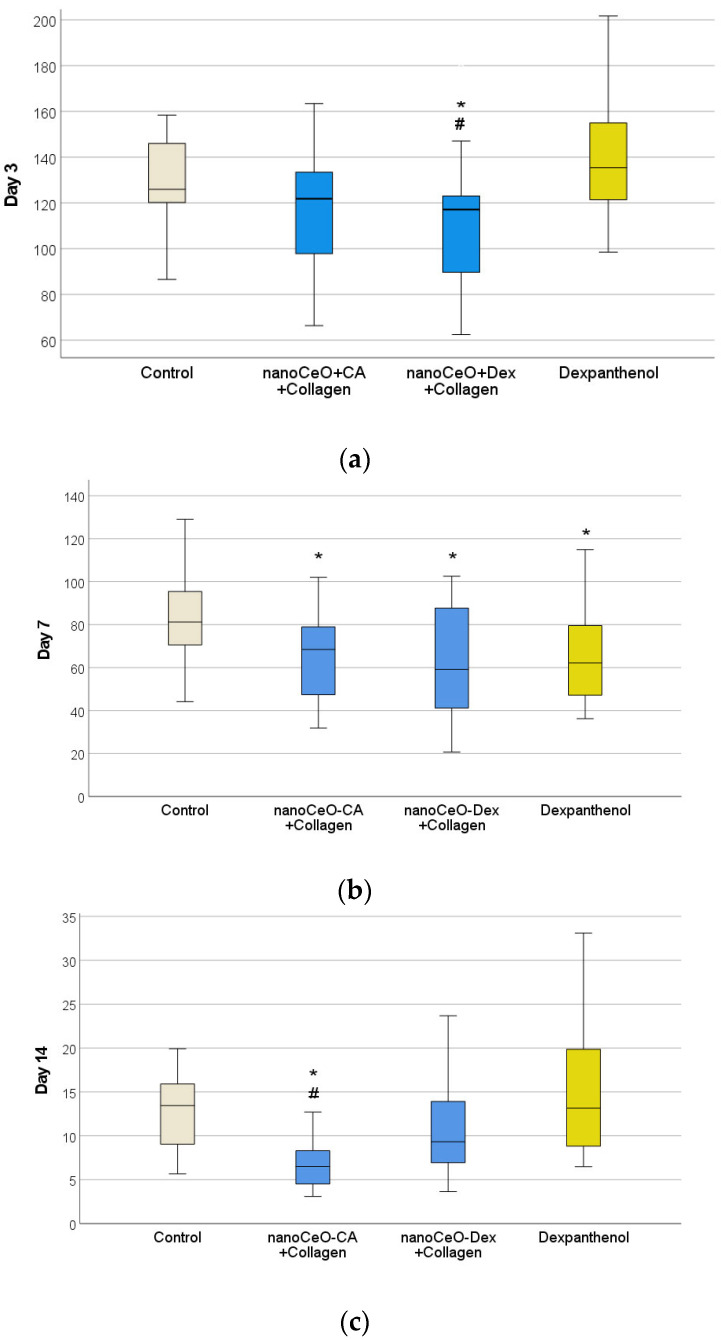
Dynamics of wound area size on days 3 (**a**), 7 (**b**), and 14 (**c**) after modeling a full-thickness skin wound (in mm^2^). (* Difference from the control group at *p* < 0.05; # Difference from the comparison group (5% Dexpanthenol ointment) at *p* < 0.05, Kruskal–Wallis test). Gray boxplots are the control, blue are the hydrogel nanocomposites under study, and yellow are the comparison group.

The study found that the most rapid positive dynamics in reducing the area of acute wounds were recorded in the groups treated with new nanodrugs. During the 1st week the best efficacy was recorded in the CeO-Dex + Collagen group (on day 3, the wound size in this group was significantly smaller than that in the control (*p* < 0.05) and comparison group (Dexpanthenol) by a factor of 1.16 (*p* < 0.05)), and by the end of the 2nd week, the best effect was established in the CeO-CA + Collagen group (on day 14, the wound size in this group was significantly smaller than that in the control group by an average factor of 2.07 (*p* < 0.05) and comparison group by a factor of 2.03 (*p* < 0.05)) (Figure 10; Table 4).

#### 3.4.2. Histological and Morphometric Examination of Wound Tissues

Analysis of the histological and morphometric images of wound tissues over time proved the efficacy and pronounced regenerative effect of the developed collagen–nanocerium composites. Differentiated cell counting in wound tissues at different healing stages, performed to assess the course of inflammation and proliferation phases, also confirmed the efficacy of nanodrugs. This was supported by a faster and more pronounced regression of leukocytes, demonstrating an anti-inflammatory effect, which was most (a trend) pronounced with CeO-Dex + Collagen, as well as a stimulatory effect on fibroblasts and their proliferation, which was most (a trend) pronounced with CeO-CA + Collagen, demonstrating accelerated regeneration (Table 5).

On day 3 after wound modeling, the wound defect volume was completely filled with granulation tissue in all the groups. Granulation tissue in the wound crater area exhibited a peak in the exudative phase of inflammation and initiation of proliferation processes. The leukocytic-necrotic layer on top of the granulations consisted of an eschar of fibrin infiltrated by polymorphonuclear leukocytes. Beneath the fibrin eschar, the formation of a layer of horizontal fibroblasts with varying degrees of maturity was visualized. The marginal epithelial ridge was absent or just beginning to form. The latter is characteristic of nanocomposite groups.

**Figure 11:**
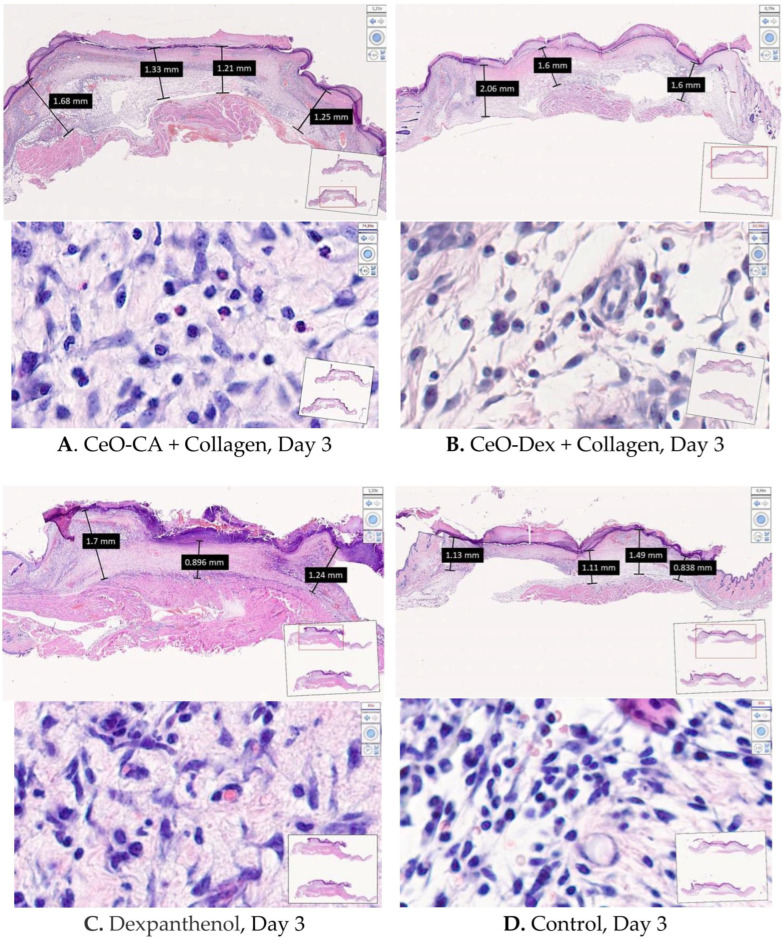
Histological image of wounds on day 3. Hematoxylin and eosin staining. (**A**) CeO-CA + Collagen, (**B**) CeO-Dex + Collagen, (**C**) Comparison drug, (**D**) Control. Overview images show a greater thickness of granulation tissue in the wound crater (indicated by the dimension lines, the size is within 0.8–2.1 mm), and more uniform filling of the wound crater with granulations in the nanocomposite groups (**A**,**B**) compared with other groups (**C**,**D**). Macroscopic images show a relatively smaller number of leukocytes in the experimental groups (**A**–**C**) compared with the control (**D**). The magnification is calculated by the software and is presented in the upper right corner of each image: (**A**)—1.21× (micro) and 74.9× times (macro); (**B**)—0.79× (micro) and 64.0× (macro); (**C**)—1.33× (micro) and 80× (macro); (**D**)—0.96× (micro) and 80× times (macro). The little picture in the lower right corner is a view of the tissue we’re examining, and the red square on it is the specific area of magnification.

**Figure 12:**
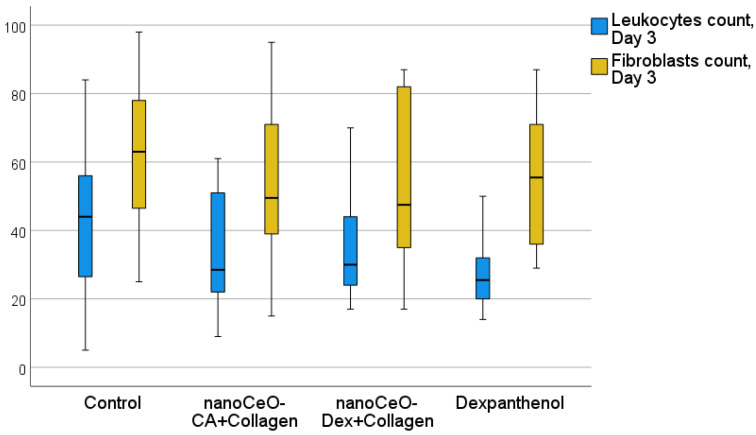
Number of leukocytic and fibroblastic cells in the wound tissues on day 3.

Morphometry of cells on day 3 revealed that the number of leukocytes in the wound tissues was lower in all treatment groups than that in the control group (on average by a factor of 1.47–1.69). This may demonstrate an early anti-inflammatory influence of all collagen nano-drugs, on par with Dexpanthenol (Figure 11 and Figure 12). The number of fibroblasts in the wounds was comparable across all the groups.

A comprehensive histological study on day 3 after wound modeling, with a comparative assessment of the state of layered organization of granulation tissue in the center and periphery of the wound defect, the presence of the marginal epithelialization ridge, features of its structure and degree of expression, and the counting and ratio of leukocytic and fibroblastic cells, allowed the compared groups to be ranked in the following order (descending): CeO-Dex + Collagen − CeO-CA + Collagen − Dexpanthenol − Control.

**Figure 13:**
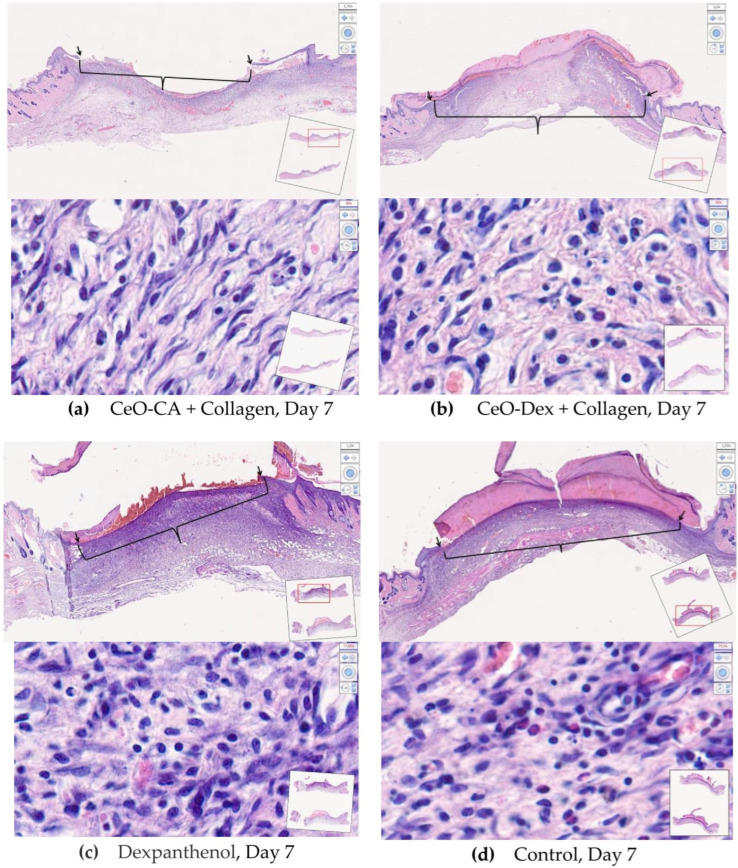
Histological picture of wounds on day 7. Hematoxylin and eosin staining. (**a**) CeO-CA + Collagen, (**b**) CeO-Dex + Collagen, (**c**) Comparison drug, (**d**) Control. (**a**,**b**) show the overview images showing the least basophilia of the regenerate, indicating greater completion of the exudative phase of inflammation compared to (**c**,**d**). Macroscopic images showing a cellular infiltrate with a predominance of fibroblastic differentiation (**a**–**c**) compared to the control group (**d**). The magnification is presented in the upper right corner of each image: (**a**)—1.38× and 80×; (**b**)—1.2× and 80×; (**c**)—1.8× and 77.7×; (**d**)—1.59× and 74.5× times. The little picture in the lower right corner is a view of the tissue we’re examining, and the red square on it is the specific area of magnification. The arrows and black curly bracket in the figure indicate the wound area.

On day 7, in all 4 study groups, the entire volume of the experimental wound cavity was filled with granulation tissue in all four study groups. Remains of the applied drug were visualized above the leukocytic-necrotic layer in the treatment groups. The leukocytic-necrotic layer consisted of an organized fibrin eschar, containing 1–2 layers of leukocytes that migrated there due to exudation. A marginal epithelial ridge of varying extents was distinguishable from the wound edges toward the center, directly under the eschar. It was formed more in the nanocomposite groups. In the control and Dexpanthenol groups, the marginal ridge was less extensive, and it was deformed or split in some wounds. The organization of the basement membrane began from the periphery toward the wound center, lagging behind the epithelial marginal ridge growth rate. The buds of the epidermal derivatives were absent. Fibroblasts of varying maturity were found in all granulation layers, but predominated in the superficial layers (Figure 13).

**Figure 14:**
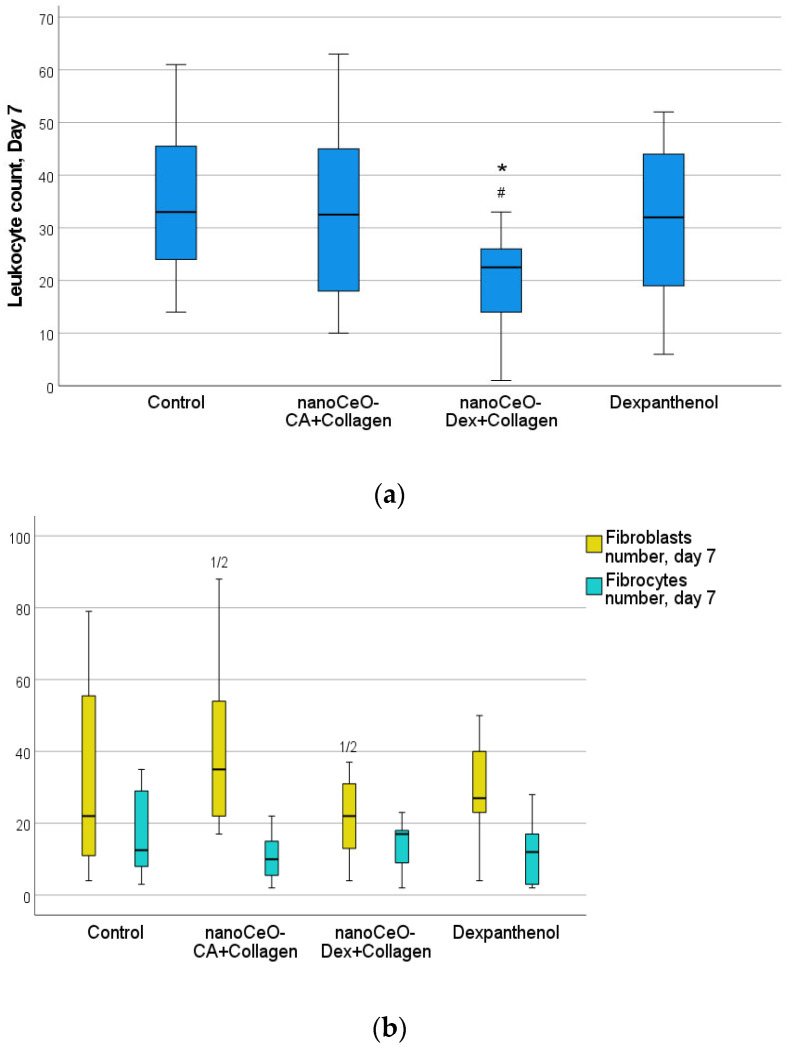
Number of leukocytic (**a**) and fibroblastic (**b**) cells on day 7 (*—significant difference from control at *p* < 0.05; #—difference from the comparison group at *p* < 0.05; 1/2—difference in the indicator between nanodrug groups 1-nanoCeO_2_-CA + Collagen and 2-nanoCeO_2_-Dex + Collagen; Kruskal–Wallis test).

Counting leukocytes in wound tissues on day 7 established significant intergroup differences, demonstrating the advantages of the CeO-Dex + Collagen group, where the number of leukocytes was 1.4 times lower than that in all other groups. A statistically significant difference was recorded only in the control and Dexpanthenol groups (*p* < 0.05); no statistically significant differences were identified in the CeO-CA + Collagen group. By day 7, cells of the fibroblastic series had formed and divided into fibroblasts and fibrocytes by maturity. There were more fibroblasts in the CeO-CA + Collagen group, on average 1.59 times more than in the CeO-Dex + Collagen group (Figure 14). The data obtained demonstrated that by day 7, the CeO-CA + Collagen drug primarily stimulated fibroblasts and their proliferation, whereas CeO-Dex + Collagen exerted a more pronounced anti-inflammatory effect.

**Figure 15:**
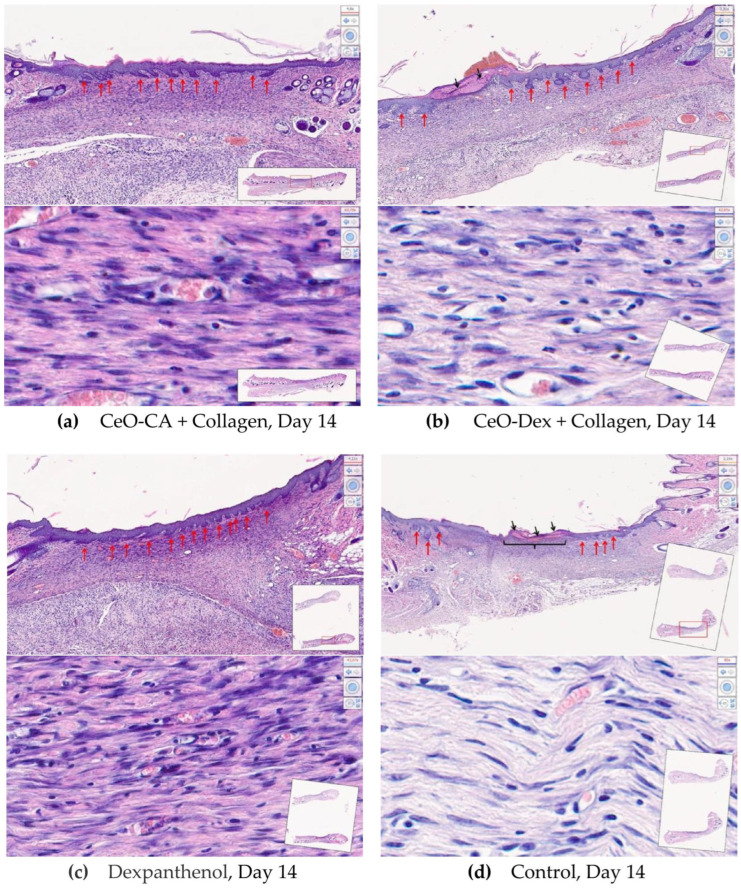
Histological picture of wounds on day 14. Hematoxylin and eosin staining. (**a**) CeO-CA + Collagen, (**b**) CeO-Dex + Collagen, (**c**) Comparison drug, (**d**) Control. Overview images show incomplete epithelialization in (**b**,**d**) (indicated by black arrows). But unlike the control group in (**b**), epithelization is visualized under the scab (blue layer). Comparable degree of differentiation and number of epidermal derivative buds in (**a**–**c**) (indicated by red arrows). Absence of growth buds in the area of incomplete epithelization in (**d**) (indicated by a black curly bracket). Macroscopic images show the greatest maturity of fibroblastic differentiation cells in (**a**,**b**) compared with (**c**,**d**). A greater number of leukocytes in the field of view in (**c**,**d**) than in (**a**,**b**). The magnification is presented in the upper right corner of each image: (**a**)—4.8× and 63.79×; (**b**)—3.31× and 62.9×; (**c**)—4.11× and 43.07×; (**d**)—2.16× and 80× times. The little picture in the lower right corner is a view of the tissue we’re examining, and the red square on it is the specific area of magnification.

On day 14 after wound modeling, the entire volume of the experimental wound cavity was filled with dense fibrous connective tissue in all animals in the CeO-CA + Collagen and CeO-Dex + Collagen groups, represented by bundles of parallel-oriented collagen fibers of varying maturity (Figure 15). The full-thickness epidermis covered the entire surface of the regenerated tissue, and all four main layers were distinguishable. Different groups showed varying degrees of epidermal derivative bud formation, without differentiation into hair follicles and sebaceous glands.

**Figure 16:**
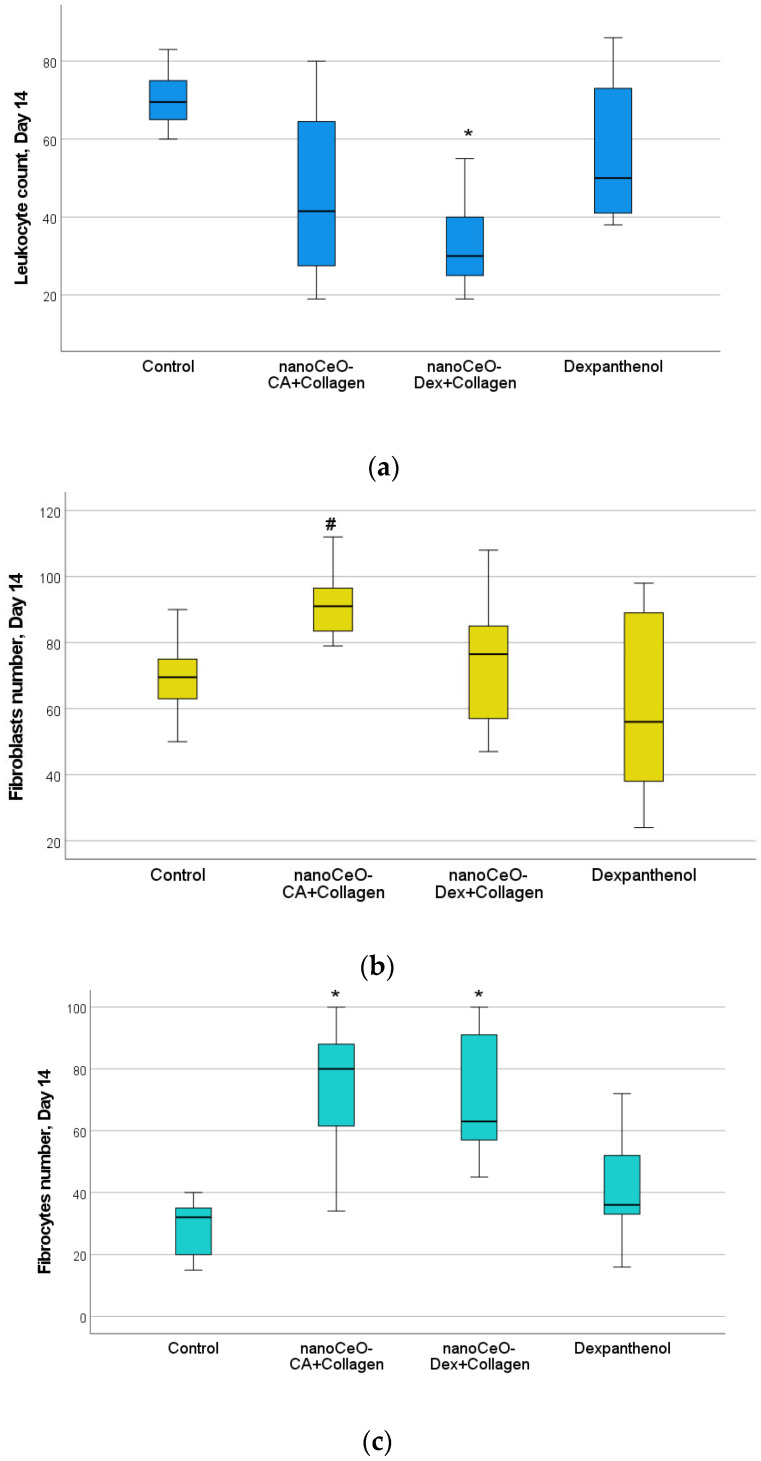
Number of leukocytes (**a**), fibroblasts (**b**), and fibrocytes (**c**) in wound tissues on day 14 of the study (*—difference from the control group at *p* < 0.05; #—difference from the comparison group at *p* < 0.05, Kruskal–Wallis test).

On day 14, statistically significant differences were recorded for all studied cell indicators (Kruskal–Wallis test, *p* < 0.05). A pronounced anti-inflammatory effect of the CeO-Dex + Collagen drug was established; the number of leukocytes in the wound tissues of this group was 2.3 times lower on average than that in the control untreated wounds (*p* < 0.05). The number of leukocytes in the CeO-CA + Collagen group on day 14 was lower than that in the control and comparison groups; however, no statistically significant differences were observed between the groups. Overall, both collagen nanodrugs stimulated fibroblasts; however, the CeO-CA + Collagen drug led to the greatest stimulation of fibroblast proliferation. The average number of fibroblasts in the wound tissues of the CeO-CA + Collagen group was, on average, 1.3 times higher than that in the control group and 1.62 times higher than that in the wounds of the comparison group treated with Dexpanthenol (*p* < 0.05). Mature fibrocytes, which are no longer capable of proliferation and whose main function is to maintain the stability of the ECM, were found at the highest concentration in the wound groups treated with the nanodrugs. The number of fibrocytes in the CeO-CA + Collagen and CeO-Dex + Collagen groups was significantly higher than that in the control: by 2.5 and 2.0 times, respectively, in the CeO-CA + Collagen and CeO-Dex + Collagen groups (*p* < 0.05). This demonstrates the completion of reparative processes and the transition to the remodeling phase by day 14 when using collagen–nanocerium composites. However, this effect was not observed in the Dexpanthenol group. The remodeling phase did not begin in any of the cases in the control group (Figure 15 and Figure 16).

Analysis of the maturity degree of the dense fibrous connective tissue of the regenerated tissue, the method of spatial organization of its fibers, the formation of epidermal layers, the degree of development, and topography of epidermal derivatives and their growth buds on day 14 after modeling the experimental wound allowed us to conclude that there were no significant differences between the compared groups and to rank them in the following descending order: CeO_2_-CA + Collagen > CeO_2_-Dex + Collagen ≥ Dexpanthenol > Control.

Thus, the study results showed that all developed collagen–nanocerium drugs significantly accelerate the wound healing process and showed better results than untreated wounds and wounds treated with Dexpanthenol. Furthermore, the anti-inflammatory effect was most pronounced for CeO-Dex + Collagen, whereas the fibroblast-stimulating effect was most pronounced for CeO-CA + Collagen.

## 4. Discussion

This study successfully led to the development of collagen–nanocerium hydrogel composites as prototypes of novel therapeutic agents for wound healing. These composites have been shown to enhance cellular metabolism and proliferation while also exhibiting antimutagenic and antioxidant properties, thereby contributing to accelerated wound regeneration in mammalian models.

The FTIR spectroscopy data revealed that the collagen-polysaccharide and protein-citrate composites based on nanocerium, synthesized by different methods (with citrate and dextran coatings), exhibited shifts in the main skeletal vibration signals (absorption bands for the composite material). Consequently, the developed nanodrug prototypes, CeO-CA + Collagen and CeO-Dex + Collagen, are bound composite materials, indicating the creation of new medicinal compounds with novel pharmacological properties distinct from those of all individual components.

The final concentrations of the main active substances were selected on the basis of a series of cell culture studies. The maximum stimulation of human fibroblast proliferation and metabolism was recorded at a nanoceria concentration of 10 < pe > ^3^ M, regardless of the synthesis method or coating type. Analysis of various CCE concentrations also demonstrated the safety and absence of cytotoxicity for all collagen concentrations used in the nanocomposite and allowed for the selection of the optimal collagen extract concentration (10%) in terms of the stimulating effect. This specific final concentration was implemented in the collagen–nanocerium composites, resulting in a 1.5-fold fibroblast stimulation. These results are consistent with data from other researchers regarding the beneficial stimulatory effect of collagen used for regeneration and wound healing [9,10,11,12,13,14,15,16,17,18,19,23,25,78].

Cerium dioxide nanoparticles at the same final concentration of 10^−3^ mol/L, in complex with 1–10% collagen, exerted an identical stimulating effect on cell metabolism (increasing it by an average of 30%), regardless of the nanoceria synthesis method and the collagen concentration in the composite. The desire to reduce the collagen concentration in the composite (from an economic standpoint, as the composite with 1% collagen showed results equivalent to 10%) was hindered by the decrease in viscosity and inconvenience of working with it in an open wound and vivarium setting. Therefore, we used a final collagen extract concentration of 10%, as we deemed the resulting viscosity of the nanocomposite more convenient for application of the regenerative drug to the skin wound surface. The viscosity could have increased with other substances such as sodium carboxymethyl cellulose, alginate, gum, or collides. However, this would introduce another substance whose effects are not always predictable, as indicated earlier [52], necessitating further investigations.

Thus, the conducted cell culture studies allowed us to select the most active and safe collagen–nanocerium complex composites—CeO (10^−3^ M)-CA + Collagen (10%) and CeO (10^−3^ M)-Dex + Collagen (10%), which significantly stimulate fibroblasts by 1.4–1.5 times (42–55%). The stimulation of fibroblasts and relative acceleration of ECM creation in the wound were expected to accelerate the regeneration process, which is why these nanodrug prototypes were selected for further study.

An important property of nanocomposites is their high antioxidant activity, prompting a separate study to determine the comparative redox activity of the synthesized nanodrugs. Bacterial-based biosensors, namely luminescent genetically modified Escherichia coli strains, were used in this study. The use of biosensors to assess the antioxidant and other biological activities of new substrates and composites is becoming increasingly widespread [82,83,84].

Studies on bacterial lux-biosensors proved the non-toxicity of the collagen–nanocerium composites, since the luminescence or cell density of the test strains did not change significantly in the first hour of exposure, and some stimulation occurred in the second hour.

Moreover, the tested samples possessed statistically significant antioxidant activities, which were most pronounced against hydrogen peroxide. The most active sample was CeO-Dex + Collagen. The application of the CeO-Dex + Collagen composite inhibited SAR formation by up to 23% (CeO-CA + Collagen, only up to 15%). The maximum antioxidant effect against hydrogen peroxide was 49% in the CeO-Dex + Collagen group and 31% in the CeO-CA + Collagen group. Therefore, CeO-Dex + Collagen possesses pronounced antioxidant properties.

It should be noted that the antioxidant effects of nanoceria have been described many times before in various models [43,47,85,86,87]. Nanoparticles of silicon dioxide used in biologically active glass, hydroxyapatite, selenium, silver, zinc oxide, titanium, and some other elements also show an antioxidant effect [88,89,90,91,92,93]. Moreover, this effect manifests itself both at the level of chemical reactions and at the level of animal and plant cells, which illustrates certain general mechanisms of nanoparticles, apparently related to the ability to use free oxygen vacancies that are easily accessible at such small sizes.

Studies on bacterial lux-biosensors have proven the non-toxicity of collagen–nanocerium composites. The nanocomposites demonstrated moderate antigenotoxic activity, protecting against up to 45% of DNA damage.

The protective properties of these compounds could be explained, first, via the unique ability of the cerium oxide nanoparticles to cycle between Ce^3+^ and Ce^4+^ oxidation states, enabling them to function as multi-enzyme mimetics that neutralize reactive oxygen species [94]. The primary mechanism involves surface oxygen vacancies that serve as active catalytic sites, where Ce^3+^ ions provide superoxide dismutase-like activity by converting O_2_^−^ radicals to H_2_O_2_, while Ce^4+^ sites exhibit catalase-like activity by decomposing H_2_O_2_ into water and oxygen. [95]. The Ce^3+^/Ce^4+^ surface ratio and oxygen vacancy concentration directly correlate with antioxidant efficacy, with smaller nanoparticles (3–5 nm) showing optimal performance owing to the higher surface area and defect density [94].

Furthermore, collagen and dextran [96,97] can act as antioxidant substrates during oxidative and genotoxic stress, providing an additional layer of protection beyond cerium nanoparticles. Collagen-derived peptides also possess antioxidant properties [98,99], which may be involved in composite biodegradation.

The superior antioxidant performance of CeO_2_-Dex + Collagen compared with that of CeO_2_-CA + Collagen can be attributed to several mechanisms. First, as shown in our previous work [51,58,59], coating influences the size of the particles, which in turn influences their activity. The dextran coating also likely enhanced the surface oxygen vacancy concentration of the nanoparticles, which served as the primary active sites for Ce^3+^-mediated ROS neutralization. The polysaccharide coating promotes an optimal Ce^3+^/Ce^4+^ surface ratio by preventing agglomeration and maintaining higher catalytic efficiency for both superoxide dismutase-like and catalase-like activities [100,101].

Furthermore, dextran may facilitate the formation of beneficial protein coronas that stabilize active surface sites, while enhancing cellular uptake pathways [102]. The significant antigenotoxic effects (up to 45% DNA protection) of these composites indicate that they operate beyond simple ROS scavenging, likely involving direct interaction with DNA-damaging molecules and activation of cellular repair mechanisms through the modulation of redox-sensitive transcription factors.

Confirmed in vitro safety and efficacy results allowed for the transition to study the efficacy of collagen–nanocerium preparations in an animal model of a full-thickness skin wound. The duration of the healing process for open acute skin wounds is primarily determined by the speed of reparative-regenerative processes, leading to a gradual reduction in the wound surface area until complete closure. Therefore, the efficacy of a wound healing drug can be determined based on the rate of decrease in the surface area of the wound. The most rapid positive dynamics in reducing the area of acute wounds were established in the nanodrug groups, which was expected based on the results of the cell culture studies with fibroblasts. While during the 1st week the best efficacy was recorded in the CeO-Dextran + Collagen group (on day 3, the wound size in this group was significantly smaller than in the control group by an average factor of 1.08 (*p* < 0.05) an than in the comparison group (Dexpanthenol)—by a factor of 1.16 (*p* < 0.05)), on day 7, the application of both nanodrugs significantly reduced the wound size relative to the control group by an average of 20–37%, without a significant intergroup difference. By the end of the 2nd week, the best effect was observed in the CeO-CA + Collagen group (on day 14, the wound size in this group was significantly smaller than that in the control group by an average factor of 2.07 (*p* < 0.05) and then in the comparison group by a factor of 2.03).

The developed nanodrugs accelerated the wound healing process and showed better results than untreated wounds and wounds treated with the registered regenerative drug starting on day 3 of treatment. Furthermore, the anti-inflammatory effect was most pronounced for CeO-Dex + Collagen, whereas the fibroblast-stimulating effect was most pronounced for CeO-CA + Collagen. The anti-inflammatory effect was accompanied by a decrease in leukocyte count at the wound edges. This phenomenon has also been corroborated by independent investigations exploring the therapeutic potential of nanoceria in the management of diverse somatic disorders [103,104,105,106].

Antioxidant effects also contribute to these processes. Oxidative stress damages cellular components, alters fibroblast metabolic pathways, and reduces their viability, whereas antioxidants reduce ROS levels, restore DNA synthesis, and increase fibroblast viability [107,108,109].

The observed temporal dynamics of the efficacy of wound healing correlated directly with the distinct antioxidant profiles of the two nanocerium composites. The best early stage performance of CeO_2_-Dex + Collagen (days 3–7) aligns with its high hydrogen peroxide-scavenging capacity (49% vs. 31% for CeO_2_-CA + Collagen). During the initial inflammatory phase, the neutrophil-derived oxidative burst generates high local H_2_O_2_ concentrations. The antioxidant activity of the nanocomposite may facilitate the transition from the inflammatory to proliferative healing phases by protecting endothelial cells from oxidative damage, thereby promoting angiogenesis and reducing inflammatory cell infiltration. On the other hand, the superior late-stage efficacy of CeO_2_-CA + Collagen (day 14) suggests that its moderate but sustained antioxidant activity (31% H_2_O_2_ scavenging, 15% SAR inhibition) may provide the deferred oxidative signaling required for fibroblast activation and ECM remodeling during tissue maturation.

The demonstrated antigenotoxic properties (up to 45% DNA protection) likely contributed to the ability of both composites to support healthy cellular proliferation and differentiation throughout the healing process, protecting against ROS-induced mutations that could compromise tissue integrity. Furthermore, the pH-responsive nature of nanoceria antioxidant activity allows these composites to adapt their protective mechanisms as the wound pH normalizes from acidic (pH 5.5–6.5) to physiological levels (pH 7.4) during healing progression, ensuring optimal ROS management at each stage.

This temporal coupling between antioxidant activity and wound healing kinetics indicates that the therapeutic efficacy of nanocerium composites is based not only on radical scavenging but also on the modulation of cellular redox homeostasis throughout the wound repair process.

We were the first to identify earlier maturation of fibrocytes and rapid creation of the ECM in the presence of the studied collagen–nanocerium composites, leading to cell differentiation in the wound, accompanied by early epithelization. Histological analysis confirmed the hypothesis that arose during cell culture studies, which showed an increased number of fibrocytes in the composite groups with advanced development of epidermal buds and the appearance of rudimentary sebaceous glands and hair follicles.

Overall, our results indicate that the application of the developed collagen–nanocerium composites for accelerating skin regeneration holds promise for use in medicine and veterinary practice. Furthermore, this study identifies new wound healing mechanisms and potentially paves the way for creating new protocols for managing patients with acute and chronic skin lesions.

## 5. Conclusions

Thus, the comprehensive interdisciplinary research allows for the following conclusions:

1. A new protein-nanocerium composite based on collagen and cerium dioxide nanoparticles coated with a polysaccharide (dextran) or a carboxylic acid (citric acid) was developed. These composites possess novel pharmacological properties, including regenerative and antioxidant effects.
2. The cell culture studies allowed for the selection of the most active and safe collagen-CeO_2_ complex composites − CeO (10^−3^ M)-CA + Collagen (10%) and CeO (10^−3^ M)-Dextran + Collagen (10%), which significantly stimulated fibroblasts by 1.5 and 1.4 times (by 42–55%), respectively, after just 72 h of co-incubation.
3. Studies on bacterial lux-biosensors have provided data on induction and protective activity for parameters such as general toxicity, antioxidant, pro-oxidant, antigenotoxic, and promutagenic activities. It was established that the nanocomposites did not demonstrate a toxic effect but possessed antigenotoxicity at a level of up to 45% as well as antioxidant activity against H_2_O_2_ (up to 49% in the CeO-Dex + Collagen group and 31% in the CeO-CA + Collagen group), thus exhibiting a protective effect.
4. Accelerated wound healing was demonstrated, starting from day 3 and continuing until day 14 (up to complete epithelialization), owing to faster ECM formation and early skin cell differentiation.
5. In models of acute skin wounds, the regenerative effect of the nanocomposites was demonstrated and exceeded that of not only the control group but also some clinical drugs, which are routinely used for skin wound healing management.

### Limitations and Prospects

Despite the substantial number of studies on nanoceria synthesis and the development of biomimetic composites, the findings presented herein do not comprehensively address all questions currently of interest to the authors. These unresolved issues pertain to the technologies employed in biomimetic fabrication, stability of the resulting composites, and their long-term efficacy and safety. This study was conducted exclusively on a single biological species, with only male subjects included in the experimental groups. Consequently, no data are available regarding the potential effects of these substances on reproductive functions. Moreover, the teratogenic potential of the developed composites was not assessed because such investigations require the use of genetically modified animal models.

Given that only an acute experimental design was employed, no data were obtained on the accumulation of nanocomposites in various organs during prolonged use, particularly within the excretory system. This consideration is especially critical in the context of chronic wound treatment, where therapeutic interventions may extend over weeks or even months.

Skin is an elastic organ that underlies the ability to move freely during physical activity. This study highlights the physiological nature of skin regeneration within the wound area, without the formation of scar-specific histological structures. However, these observations were limited to the acute phase. Data on the tensile strength of regenerated skin and the presence or absence of scar formation over the long term have not yet been obtained and remain the focus of ongoing research. Although any cosmetic defect at the regeneration site may be of minor concern to general surgeons, it holds greater relevance for specialists in aesthetic and plastic surgery.

In light of these considerations, the authors have not initiated clinical trials at this stage because of concerns regarding potential toxicity or other adverse effects. Therefore, the conclusions drawn are deliberately cautious. Clinical testing of the nanocomposites is scheduled to begin within a year, contingent upon the successful completion of the aforementioned studies verifying the safety and efficacy of the nanoceria-collagen composite. If these results are favorable, the developed composites may represent a significant advancement among current wound-healing agents.

## Figures and Tables

**Table 1:** Methods of collagen–nanocerium composites’ preparation.

CeO_2_ and Collagen Content in the Final Concentration of the Prototype Drug	Nanoparticles (CeO-CA, CeO-Dex)	Collagen-Containing Extract, mL	Phosphate Buffer (mL)	Total Volume (mL)
CeO_2_ (10^−3^ M) + Collagen (10%)	10^−2^ M—10 mL	10	80	100
CeO_2_ (10^−3^ M) + Collagen (1%)	10^−2^ M—10 mL	1	89	100
CeO_2_ (10^−4^ M) + Collagen (10%)	10^−3^ M—10 mL	10	80	100
CeO_2_ (10^−4^ M) + Collagen (1%)	10^−3^ M—10 mL	1	89	100

**Table 2:** Research groups of hydrogel nanocomposites.

No	Collagen	CeO-CA NPs	CeO-Dex NPs
1	10%	10^−3^ M	–
2	10%	10^−4^ M	–
3	10%	–	10^−3^ M
4	10%	–	10^−4^ M
5	1%	10^−3^ M	–
6	1%	10^−4^ M	–
7	1%	–	10^−3^ M
8	1%	–	10^−4^ M

**Table 3:** The strains of luminescent lux bacterial biosensors used in the study.

*E. coli* Strains	Detectable Change Type	Measurable Factor
MG1655 pXen7	Decrease	Toxicity
MG1655 pKatG	Increase/Decrease	Prooxidant/antioxidant activity (hydrogen peroxide)
MG1655 pSoxS	Increase/Decrease	Prooxidant/antioxidant activity (superoxide anion radical)
MG1655 pRecA	Increase/Decrease	Antigenotoxic/promutagenic activity

**Table 4:** Dynamics of the wound area size on days 1, 3, 7, and 14 of the study, mm^2^.

	Control (C)	CeO-CA + Collagen + (1)	CeO-Dex + Collagen (2)	Dexapantenol (D)	*p*-Value (Total)	*p*-Value with the Bonferroni Correction
Day 1	144,7 [128.3, 170.3]	143,1 [121.9, 158.1]	141,9 [116.1, 161.7]	149,2 [137.2, 173.0]	0.328	
Day 3	125.9 [120.2, 146.0]	121.8 [97.9, 133.8]	117.1 [89.7, 123.0]	135.4 [121.4, 155.0]	0.003 C/2 * D/2 * D/1 *	D/2 (0.003)
Day 7	81.2 [70.6, 95.5]	68.4 [47.4, 78.9]	59.1 [41.2, 87.7]	62.2 [47.2, 79.6]	0.041 C/1 * C/2 * C/D *	K/2 (0.042)
Day 14	13.5 [9.06, 15.9]	6.5 [4.5, 8.3]	9.3 [6.9, 13.9]	13.2 [8.8, 19.9]	0.004 C/1 * D/1 *	K/1 (0.022) D/1 (0.006)

* The results are presented as the median (first row) and [upper (lower] quartile values (2nd row). Bonferroni correction for multiple tests was applied to the significance values.

**Table 5:** Differentiated counting of leukocytic and fibroblast cells of different degrees of maturity in wound tissues on days 3, 7, and 14 in the study groups.

Day	Number of Cells in the	Control (C)	CeO-CA + Collagen + (1)	CeO-Dex + Collagen (2)	Dexapantenol (D)	*p*-Value (Total)	*p*-Value with the Bonferroni Correction
3	Leukocytes	44 [26, 56]	29 [22, 51]	30 [24, 44]	26 [20, 32]	0.067	
3	Fibroblasts	63 [46, 78]	49 [39, 71]	48 [35, 82]	56 [36, 71]	0.314	
7	Leukocytes	33 [24, 46]	32 [18, 45]	23 [14, 26]	32 [19, 44]	0.008 *	C/2, 2/D
7	Fibrocytes	13 [8, 29]	10 [6, 15]	17 [9, 18]	12 [3, 17]	0.745	
7	Fibroblasts	23 [11, 56]	35 [22, 54]	22 [13, 31]	27 [23, 40]	0.036 *	1/2
14	Leukocytes	69 [64, 75]	42 [27, 64]	30 [24, 41]	51 [41, 73]	0.029 *	C/2
14	Fibrocytes	32 [20, 35]	79 [61, 88]	63 [57, 91]	36 [33, 52]	0.005 *	C/1, C/2
14	Fibroblasts	70 [63, 76]	91 [83, 97]	77 [57, 85]	56 [38, 89]	0.049 *	1/D

* The results are presented as the median (first row) and [upper (lower)] quartile values (2nd row).

## Data Availability

The data is available in this manuscript and from the corresponding author.

## Abbreviations

The following abbreviations are used in this manuscript:

CCE	Collagen-Containing Extract
ECM	extracellular matrix
E. coli	Escherichia coli
FTIR	Fourier-transform infrared spectroscopy
IC	induction coefficient
OD	optical density
PBS	phosphate-buffered saline
RLU	relative light units
ROS	reactive oxygen species
SAR	superoxide anion radical
